# Cu(II) and Ni(II) Complexes with New Tridentate NNS Thiosemicarbazones: Synthesis, Characterisation, DNA Interaction, and Antibacterial Activity

**DOI:** 10.1155/2019/3520837

**Published:** 2019-07-01

**Authors:** Dorian Polo-Cerón

**Affiliations:** Departamento de Química, Universidad Del Valle, Calle 13 No. 100-00, Cali (76001000), Colombia

## Abstract

This paper reports the synthesis and detailed characterisation of copper(II) and nickel(II) complexes with tridentate thiosemicarbazone ligands **H**_**2**_ **L1** and **H**_**2**_ **L2** derived from 2-acetylpyrazine. The ligands and their metal complexes were characterised by different physicochemical techniques, including elemental and thermogravimetric analysis; UV-Vis, IR, ^1^H-NMR, and ^13^C-NMR spectroscopy; molar conductance measurements; and mass spectrometry. The crystal structure of the **H**_**2**_ **L1** ligand was determined by single crystal X-ray diffraction studies. The spectral data showed that the thiosemicarbazone behaves as an NNS tridentate ligand through the nitrogen atoms of the azomethine group and pyrazine ring and the sulphur atom of the thioamide group. Elemental and thermal analyses indicated that the obtained metal complexes had a 1 : 1 stoichiometry (metal-ligand). The interactions between these complexes and calf thymus DNA (CT-DNA) were studied by electronic absorption and viscosity measurements. The activities of these compounds against oxidative DNA cleavage were examined by agarose gel electrophoresis. Cu(II) and Ni(II) complexes can wind DNA strands through groove interactions and promote strand breakage of the plasmid pmCherry under oxidative stress conditions. Moreover, all the complexes could interact more strongly with DNA than could with the free ligands. Finally, the antibacterial activities of the ligands and their complexes were determined by *in vitro* tests against Gram-positive bacterial strains (*S. aureus* ATCC 25923, *L. monocytogenes* ATCC 19115, and *B. cereus* ATCC 10876) and Gram-negative bacterial strains (*E. coli* ATCC 25922, *S. typhimurium* ATCC 14028, and *K. pneumoniae* ATCC BAA-2146) using the broth microdilution method. The metal complexes showed greater antimicrobial activities than the precursor ligands against some of the microorganisms.

## 1. Introduction

In recent decades, coordination compounds with transition metals have become quite important in medicinal chemistry [1–4]. The fight against infectious diseases, one aspect of this field, has seen great advances; however, antimicrobial resistance remains a major obstacle and continues to increase, and it is now considered a global public health problem [5]. Multiple investigations have been performed to test new free organic or metal coordination compounds, which can be precursors of more efficient and less toxic drugs by acting through different biological mechanisms [6–8].

Thiosemicarbazones (TSCs) are organic compounds with the structure R^1^R^2^C = N-NH-(C=S)-NR^3^R^4^, which have been used as potential antituberculosis agents as early as 1950s [9]. Subsequently, and due to their potential therapeutic properties, these multidentate ligands have constituted an important class of compounds whose properties serve in a wide range of uses [10, 11]. The electronic properties of the NNS donor system and the variety of chemical species that the system can produce are the reasons why TSC ligands act as good chelating agents that can easily coordinate with a great variety of transition metal ions, forming complexes that can change the biological activity of precursor ligands [12]. TSCs and their transition metal complexes, due to their chemical versatility, have a wide spectrum of pharmacological properties, such as antibacterial, antifungal, antiparasitic, and antiviral [13–16]. The interaction between these compounds and DNA has attracted great attention in medicinal chemistry due to the potential use of these complexes as antineoplastic agents [17]. The cytotoxic activities of these ligands are improved upon coordination of metal ions, such as Cu, Ni, Zn, and Pd, which can also improve their lipophilicity and mechanism of action within the cell [18].

Considering the pharmacological potential of Cu(II) and Ni(II) thiosemicarbazone complexes and following our interest in developing new molecules with biological activity [2], this paper describes the synthesis and characterisation of Cu(II) and Ni(II) complexes with new *N*(4)-(4-R-phenyl)thiosemicarbazones derived from 2-acetylpyrazine. This study examines how these compounds interact with DNA strands through different experiments, in addition to testing their antibacterial activities against Gram-positive and Gram-negative bacterial strains.

## 2. Materials and Methods

### 2.1. General Information

All glassware was completely dried in an oven at 100°C for 12 h. Chemical reagents and solvents were purchased from commercial suppliers. Melting points were determined in an automatic OptiMelt MPA100 device and reported uncorrected. The ^1^H-NMR and ^13^C-NMR spectra were recorded in DMSO-*d*_6_ at 25°C on a Bruker Avance II 400 MHz Ultrashield spectrometer; chemical shifts (*δ*) are expressed in ppm using tetramethylsilane (TMS) as an internal standard. Complete assignment of the NMR signals of the new compounds was supported by two-dimensional spectral analysis (heteronuclear multiple bond correlation (HMBC) and heteronuclear single quantum correlation (HSQC)). Thermal analyses were performed using a thermogravimetric analyser (TGA 550, TA Instruments, USA) under a nitrogen atmosphere. Electron ionisation mass spectrometry (EI-MS) spectra were recorded on a Shimadzu GCMS-QP2010 spectrometer (Shimadzu, Japan) operated in the electron impact ionisation mode at 40 eV. Infrared spectra from 4000 cm^−1^ to 600 cm^−1^ were recorded on a Shimadzu Affinity 1 spectrometer (FT-IR) equipped with an attenuated total reflection (ATR) accessory; the spectra were taken in KBr pellets. Elemental analyses were performed using a Flash EA 1112 CHN analyser (Thermo Fisher Scientific, USA). The percentage of metals was measured in duplicate by atomic absorption spectrometry (iCE 3000 series AA spectrometer) using the flame method. The molar conductance values of 10^−3^ mol·L^−1^ solutions of the complexes in DMSO were measured with an Orion™ 131S Basic Waterproof Conductivity Meter (Thermo Fisher Scientific, USA) using a 0.01 mol·L^−1^ aqueous KCl solution for calibration. The UV-Vis spectroscopy studies were performed on a Jasco V-630 BIO UV-visible spectrometer equipped with PAC-743 model temperature control accessory. The metal-ligand binding ratio was investigated using Job's method. 2% DMSO solutions of TSCs and metal ions were prepared with increasing mole ratios of TSC : metal ion from 1 : 0 to 0 : 1. Viscosity measurements were carried out using a Cannon-Ubbelohde Semi-Micro Viscometer (size 75).

### 2.2. Synthesis of Ligands and Coordination Compounds

*2-Acetylpyrazine N(4)-phenylthiosemicarbazone (* ***H***_***2***_ ***L1***). An ethanolic solution (3 mL) of aniline (0.55 mol·L^−1^, 1.64 mmol) and potassium hydroxide (0.55 mol·L^−1^, 1.64 mmol) was slowly added to 2 mL (33.1 mmol, excess) of carbon disulphide in an ice bath (4°C). Two phases were immediately obtained, and they were kept under constant stirring for 24 hours at room temperature. After the reaction was complete, a white solid was observed in the reaction mixture. Hydrazine monohydrate (80 *μ*L, 1.65 mmol) was added, and the mixture was refluxed at 80°C for 4 additional hours. Subsequently, the solution was concentrated to 3 mL and cooled to 4°C in an ice bath. 5 mL of a cold 1 : 1 mixture of hexane and dichloromethane was added to precipitate *N*(4)-phenylthiosemicarbazide. The solid was then isolated by filtration and washed with 8 mL of cold hexane.

A methanolic solution (2.5 mL) of 2-acetylpyrazine (0.48 mol·L^−1^, 1.20 mmol) and three drops of glacial acetic acid were added to a solution (2.5 mL) of *N*(4)-phenylthiosemicarbazide (0.48 mol·L^−1^, 1.20 mmol); the system was refluxed at 70°C for 2 hours. After the reaction, a pale-yellow solid (H_2_L_1_) was observed; excess solvent was removed by evaporation under reduced pressure, and the product was washed with cold hexane (3 × 8 mL). Pale-yellow powder (yield: 276.4 mg, 85%). m.p.: 206–207°C. C_15_H_13_N_5_S, elemental analysis; C, 57.12 (calc. 57.54); H, 4.75 (4.83); N, 25.16 (25.81); and S, 10.75 (11.82)%. IR (ATR cm^−1^) ν: 3303 m, 3214 m, 1615 w, 1587 m, 1528 s, 1516 s, 1490 s, 1467 s, 1442 s, 1408 m, 1361 m, 1302 m, 1259 m, 1189 m, 1167 s, 1153 m, and 854 s. ^1^H-NMR (DMSO-*d*_6_, 400 MHz): *δ*_H_ 2.45 (s, 3H, H8), 7.24 (t, *J* = 7.4 Hz, 1H, H16), 7.39 (t, *J* = 7.8 Hz, 2H, H15), 7.54 (d, *J* = 7.5 Hz, 2H, H14), 8.62 (d, *J* = 2.6 Hz, 1H, H2), 8.64 (dd, *J* = 2.6 Hz, 1.5 Hz, 1H, H3), 9.78 (d, *J* = 1.2 Hz, 1H, H5), 10.32 (s, 1H, H12), and 10.81 (s, 1H, H10). ^13^C-NMR (DMSO-*d*_6_, 100 MHz): *δ*_C_ 12.2 (C8), 125.6 (C16), 126.4 (C14), 128.0 (C15), 139.1 (C13), 143.1 (C3), 143.5 (C5), 144.1 (C2), 147.1 (C7), 149.9 (C6), and 177.4 (C11). MS electron impact (*m*/*z*, 40 eV): 271 [M^+^]. *λ*_Abs_ = 326 nm, 204 nm. *λ*_Em_ = 339 nm, 301 nm. *λ*_Ex_ = 226 nm.

*2-Acetylpyrazine N(4)-(4-chlorophenyl)thiosemicarbazone (* ***H***_***2***_ ***L2***). An ethanolic solution (3 mL) of 4-chloroaniline (0.67 mol·L^−1^, 2.00 mmol) and potassium hydroxide (0.67 mol·L^−1^, 2.00 mmol) was slowly added to 2 mL (33.1 mmol, excess) of carbon disulphide in an ice bath (4°C); two phases were immediately obtained, and they were kept under constant stirring for 24 hours at room temperature. After the reaction was complete, a white solid was observed in the reaction mixture. Hydrazine monohydrate (97 *μ*L, 2.00 mmol) was added, and the mixture was refluxed at 80°C for 4 hours. Then, the solution was concentrated to 3 mL and cooled to 4°C in an ice bath; 5 mL of a cold 1 : 1 mixture of hexane and dichloromethane was added to precipitate *N*(4)-(4-chlorophenyl)thiosemicarbazide; the solid was isolated by filtration and washed with 8 mL of cold hexane.

A methanolic solution (2.5 mL) of 2-acetylpyrazine (0.56 mol·L^−1^, 1.39 mmol) and three drops of glacial acetic acid were added to a solution (2.5 mL) of *N*(4)-(4-chlorophenyl)thiosemicarbazide (0.56 mol·L^−1^, 1.39 mmol); the system was refluxed at 70°C for 2 hours. After the reaction time, a pale-yellow solid (**H**_**2**_ **L2**) was observed; excess solvent was removed by evaporation under reduced pressure, and the product was washed with cold hexane (3 × 8 mL). Pale-yellow powder (yield: 347.6 mg, 82%). m.p.: 207–209°C. C_15_H_12_N_5_SCl, elemental analysis; C, 50.62 (calc. 51.06); H, 3.87 (3.96); N, 22.77 (22.90); and S, 10.12 (10.48)%. IR (ATR cm^−1^) ν: 3283 w, 3186 w, 3054 w, 1615 w, 1590 m, 1539 s, 1501 m, 1488 m, 1465 m, 1396 m, 1356 m, 1315 m, 1303 m, 1279 w, 1248 m, 1187 m, 1164 m, 1152 m, and 851 m. ^1^H-NMR (DMSO-*d*_6_, 400 MHz): *δ*_H_ 2.45 (s, 3H, H8), 7.44 (t, *J* = 8.7 Hz, 2H, H14), 7.58 (d, *J* = 8.7 Hz, 2H, H15), 8.63 (d, *J* = 2.5 Hz, 1H, H2), 8.64 (d, *J* = 2.5 Hz, 1H, H3), 9.77 (d, *J* = 1.3 Hz, 1H, H5), 10.32 (s, 1H, H12), and 10.93 (s, 1H, H10). ^13^C-NMR (DMSO-*d*_6_, 100 MHz): *δ*_C_ 12.3 (C8), 128.0 (C15), 128.1 (C14), 129.7 (C16), 138.1 (C13), 143.1 (C3), 143.5 (C5), 144.1 (C2), 147.4 (C7), 149.8 (C6), and 177.5 (C11). MS electron impact (*m*/*z*, 40 eV): 307 [M^+^]. *λ*_Abs_ = 326 nm, 204 nm. *λ*_Em_ = 339 nm, 301 nm. *λ*_Ex_ = 226 nm.

*[Cu(H*_*2*_ *L1)(NO*_*3*_ *)]NO*_*3*_ *·3H*_*2*_ *O (* ***1*** *).* A methanolic solution (5 mL) of Cu(NO_3_)_2_·3H_2_O (0.074 mol·L^−1^, 0.37 mmol) was slowly added to a methanolic solution (15 mL) of 2-acetylpyrazine *N*(4)-phenylthiosemicarbazone (0.025 mol·L^−1^, 0.37 mmol), and the formation of a precipitate was observed. The mixture was kept under constant stirring for 5 hours at 70°C. Finally, the dark brown solid was isolated by filtration, washed with 5 mL of cold methanol, and then dried in a desiccator for 2 days. Deep brown powder (yield: 111.2 mg, 65%). m.p.: 231–233°C. C_13_H_19_CuN_7_O_9_S, elemental analysis, %; C, 30.28 (calc. 30.44); H, 3.35 (3.73); N, 18.88 (19.12); S, 6.14 (6.25); and Cu, 12.19 (12.39). IR (KBr pellet cm^−1^) ν: 3308 s, 3138 m, 3072 w, 1559 m, 1544 m, 1478 m, 1434 m, 1306 m, 1280 s, 1253 m, 1147 s, 1091 m, 1008 s, 842 w, 750 s, and 692 m. TGA mass loss: 10.8% (50–200°C, 1 step, calc. 3 × H_2_O = 10.5%) and 62.1% (220–250°C, 1 step, calc. Cu(NO_3_)_2_ formation = 63.4%). Λ (DMSO, 20°C) (*μ*S·cm^−1^): 29.7.

*[Cu(H*_*2*_ *L2)(NO*_*3*_ *)]NO*_*3*_ *·H*_*2*_ *O (* ***2***). A methanolic solution (5 mL) of Cu(NO_3_)_2_·3H_2_O (0.065 mol·L^−1^, 0.33 mmol) was slowly added to the methanolic solution (15 mL) of 2-acetylpyrazine *N*(4)-(4-chlorophenyl)thiosemicarbazone (0.021 mol·L^−1^, 0.33 mmol), and the formation of a precipitate was observed. The mixture was kept under constant stirring for 5 hours at 70°C. Finally, the dark brown solid was filtered and washed with 5 mL of cold methanol and then dried in a desiccator for 2 days. Deep brown powder (yield: 111.0 mg, 68%). m.p.: 239–240°C. C_13_H_14_ClCuN_7_O_7_S, elemental analysis, %; C, 32.03 (calc. 30.54); H, 3.10 (2.76); N, 20.31 (19.17); S, 5.32 (6.27); and Cu, 12.56 (12.43). IR (KBr pellet cm^−1^): 3299 m, 3183 w, 3063 w, 1595 m, 1541 m, 1477 m, 1447 m, 1310 m, 1282 s, 1248 m, 1147 m, 1082 s, 1040 w, 1008 s, 834 m, 803 m, and 688 w. TGA mass loss 3.89% (50–200°C, 1 step, calc. 1 × H_2_O = 3.52%) and 62.7% (230–270°C, 1 step, calc. Cu(NO_3_)_2_ formation = 63.3%). Λ (DMSO, 20°C) (*μ*S·cm^−1^): 24.5.

*[Ni(H*_*2*_ *L1)(NO*_*3*_ *)]NO*_*3*_ *·CH*_*3*_ *OH (* ***3***). Compound **3** was prepared following the same procedure than the synthesis of compound **1**. Brown powder (yield: 93.1 mg, 55%). m.p.: >300°C. C_14_H_17_N_7_NiO_7_S, elemental analysis, %; C, 35.13 (calc. 34.59); H, 3.28 (3.53); N, 19.31 (20.17); S, 7.90 (6.60); and Ni, 12.26 (12.07). IR (KBr pellet cm^−1^) ν: 3344 m, 3073 w, 1596 w, 1540 w, 1509 w, 1467 s, 1435 s, 1316 m, 1280 s, 1249 m, 1148 s, 1087 m, 1023 m, 843 m, 755 s, 694 m, and 640 m. TGA mass loss 6.11% (30–120°C, 1 step, calc. 1 × CH_3_OH = 6.59%) and 50.8% (250–500°C, 2 step, calc. Ni(NO_3_)_**2**_ formation = 49.7%). Λ (DMSO, 20°C) (*μ*S·cm^−1^): 25.3.

*[Ni(H*_*2*_ *L2)(NO*_*3*_ *)]NO*_*3*_ (***4*** *):* Compound **4** was prepared following the same procedure than the synthesis of compound **2**. Brown powder (yield: 79.2 mg, 49%). m.p.: >300°C. C_13_H_12_ClN_6_NiO_3_S, elemental analysis, %; C, 32.88 (calc. 31.96); H, 2.71 (2.48); N, 19.50 (20.07); S, 6.98 (6.56); and Ni, 11.86 (12.02). IR (KBr pellet cm^−1^) ν: 3335 m, 3064 m, 1592 w, 1536 m, 1501 s, 1480 m, 1450 s, 1391 m, 1306 m, 1271 s, 1248 m, 1148 s, 1078 s, 1005 m, 830 s, 816 m, 747 m, and 685 w. TGA mass loss 47.7% (280–320°C, 1 step, calc. Ni(NO_3_)_**2**_ formation = 49.9%). Λ (DMSO, 20°C) (*μ*S·cm^−1^): 26.1.

### 2.3. Single-Crystal Structure Determination

Selected crystallographic data are presented in Table 1. A suitable crystal of **H**_**2**_ **L1** was mounted on a glass fibre and used for data collection on a Bruker SMART diffractometer equipped with an APEX CCD area detector. Frames were collected by omega scans and integrated with the Bruker SAINT software package using the appropriate unit cell [19]. The structure was solved using the SHELXS-97 program [20] and refined by full-matrix least-squares on *F* ^2^ with SHELXL-97 [21]. Weighted *R* factors, *R*_w_, and all goodness-of-fit indicators, S, were based on *F* ^2^.

CCDC 1861232 contains the supplementary crystallographic data for **H**_**2**_ **L1**. These data can be obtained free of charge via http://www.ccdc.cam.ac.uk/structures/ or from the Cambridge Crystallographic Data Centre, 12 Union Road, Cambridge CB2 1EZ, UK; fax: (+44) 1223-336-033; or e-mail: deposit@ccdc.cam.ac.uk.

### 2.4. DNA Interaction

DNA interaction studies with the obtained compounds were performed via electronic absorption experiments. The oxidative cleavage was monitored by agarose gel electrophoresis, and the viscosity measurements were performed following standard methodologies and procedures modified by our laboratory [22, 23]. Lyophilised DNA from calf thymus (CT-DNA), obtained from Sigma Aldrich, and the pmCherry vector extracted from *E. coli* BL21 (DH5*α*) were used. All DNA solutions had an A_260_/A_280_ ratio between 1.8 and 1.9, indicating that the DNA was free of RNA and proteins. The DNA was resuspended in 10 mmol·L^−1^ Tris and 1 mmol·L^−1^ EDTA in deionised water with the pH adjusted to approximately 7.5. The DNA solutions were stored at −5°C.

### 2.5. UV-Vis Spectroscopy Studies

Electronic absorption spectra were recorded on a Jasco V-630 BIO UV-visible spectrometer equipped with a PAC-743 model temperature control accessory. Titrations were performed at 20°C, and the concentration of compound remained constant (50 *μ*mol·L^−1^), while the concentration of CT-DNA was gradually increased (0 to 50 *μ*mol·L^−1^). After each addition of titrant, an electronic absorption spectrum of the test solution was recorded from 230 nm to 500 nm. The solutions of the complexes contained deionized water and DMSO of approximately 0.1%. The CT-DNA absorbance was eliminated by adding an equal amount of CT-DNA to the sample and the standard solution. A solution of CT-DNA (2100 *μ*mol·L^−1^) in nucleotides was prepared using *ε*_260_ = 6600 cm^−1^·mol^−1^·L as the molar extinction coefficient, and the absorbance was measured at 260 nm. The stock solution was stored at −5°C.

### 2.6. Viscosity Studies

Viscosity measurements were carried out using a Cannon-Ubbelohde Semi-Micro Viscometer (size 75), thermostated in a water bath maintained at 22.0 ± 0.1°C. The relative viscosities of five DNA solutions were determined; the solutions had the same concentrations of CT-DNA (600 *μ*M) but increasing amounts of the test compound (*R* = [compound]/[DNA]) between 0.2 and 2). The efflux times were recorded by processing the video's photograms with Camtasia Studio® software.

### 2.7. Electrophoresis Studies

Oxidative cleavage catalysed by the complexes was studied by incubating different solutions, 15 *μ*L each, containing constant concentrations of H_2_O_2_ and the pmCherry vector extracted from *E. coli* BL21(DH5*α*) with varying concentrations of the complexes in DMSO. Samples were incubated under physiological conditions (37°C and pH = 7.0) for 90 minutes in an Eppendorf ThermoMixer® C. After incubation, the samples were loaded on 1% agarose gels with 1X TAE buffer and Bioline HyperLadder™ 1 kb as the molecular weight marker. For the electrophoresis runs, a Thermo Scientific™ Owl™ EasyCast™ B1 Mini Gel electrophoresis system and GelGreen™ dye were used to visualise the gel in a MaestroGen UltraBright Led 470 nm transilluminator.

### 2.8. Antibacterial Activity Tests

The antibacterial activities of all of the compounds were determined for three Gram-positive bacterial strains (*S. aureus* ATCC 25923, *L. monocytogenes* ATCC 19115, and *B*. *cereus* ATCC 10876) and three Gram-negative bacterial strains (*E. coli* ATCC 25922, *S. typhimurium* ATCC 14028, and *K. pneumoniae* ATCC BAA-2146). Standard procedures involving the microdilution technique recommended by the Clinical and Laboratory Standards Institute (CLSI) were used to determine the antimicrobial susceptibility [24]. The minimum inhibitory concentrations (MICs) were evaluated in a concentration range from 2000 to 3.9 *μ*g·mL^−1^. The negative control was Mueller–Hinton broth (MHB) with no bacteria, and the positive control was MHB with only bacteria. All determinations were performed in triplicate. Ciprofloxacin (range between 4.0 and 0.008 *μ*g·mL^−1^) and AgNO_3_ (range between 500 and 100 *μ*g·mL^−1^) were used as standard bactericides. To exclude the possibility that resistance could be induced by ciprofloxacin and AgNO_3_, the antimicrobial susceptibility of isolates was tested by the broth microdilution method on the antibacterial agent-free medium. An additional study was performed to test the effect of the solvent in the biological screening, and DMSO was found to have no activity against any of the tested strains.

## 3. Results and Discussion

### 3.1. Synthesis and Characterisation of Ligands

**Scheme 1:**
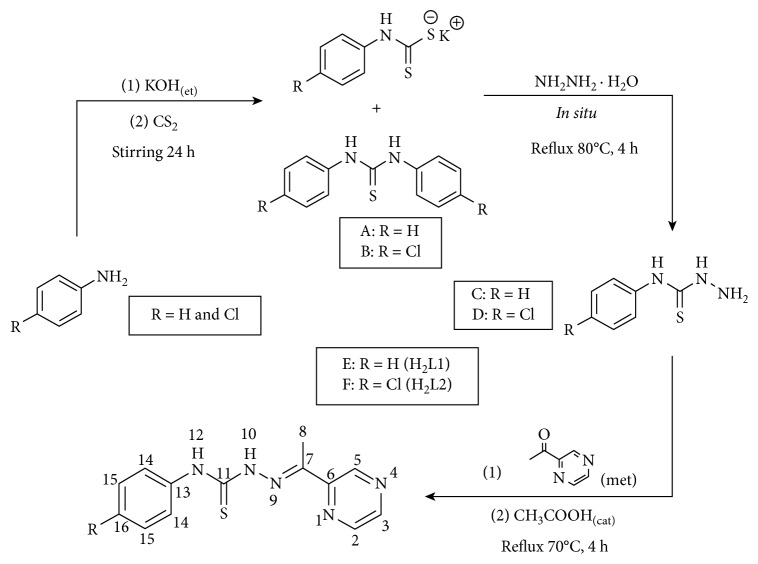
Synthesis of thiosemicarbazones from 2-acetylpyrazine.

The syntheses of 2-acetylpyrazine *N*(4)-phenylthiosemicarbazone (**H**_**2**_ **L1**) and 2-acetylpyrazine *N*(4)-(4-chlorophenyl)thiosemicarbazone (**H**_**2**_ **L2**) were performed through a three-step synthetic route (Scheme 1), following previously reported standard methodologies [14, 25, 26]. First, the nucleophilic addition of the *p*-substituted aniline to carbon disulphide occurred to generate the respective salt of the dithiocarbamate. The compounds that precipitated during this first step were identified as 1,3-diphenylthiourea (Scheme 1(A)) (MS electron impact (*m*/*z*, 40 eV), 228 [M^+^]) and 1,3-bis(4-chlorophenyl)thiourea (Scheme 1(B)) (MS electron impact (*m*/*z*, 40 eV), 297 [M^+^]). These compounds were precursors for the synthesis of the thiosemicarbazides (Schemes 1(C) and 1(D)) via substitution with hydrazine monohydrate. In addition to the determination of the melting points of *N*(4)-phenylthiosemicarbazide (m.p.: 140–141°C) and *N*(4)-(4-chlorophenyl)thiosemicarbazide (mp: 165–167°C), the characteristic infrared bands of each compound were assigned in accordance with previous reports [14, 27, 28]. The condensation of the latter compounds with 2-acetylpyrazine, followed by acid hydrolysis, afforded the TSCs as crystalline solids in greater than 80% yields (Schemes 1(E) and 1(F)). The elemental analysis results (C, H, and N), one- and two-dimensional NMR spectroscopic analyses, and electron impact mass spectrometry measurements confirmed the formation of the proposed products (Scheme 1). The crystal structure of ligand **H**_**2**_ **L1** was determined by single crystal X-ray diffraction studies, confirming the structure elucidated by the spectroscopic data.

#### 3.1.1. Nuclear Magnetic Resonance Spectroscopy

**Figure 1:**
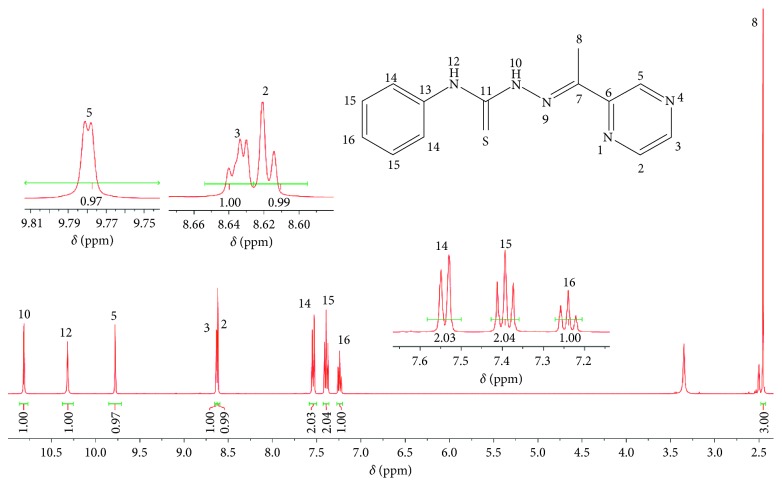
^1^H-NMR spectrum for H_2_L1.

The recorded signals obtained in the ^1^H and ^13^C in NMR spectra are in agreement with the proposed structures of the TSCs. In the ^1^H-NMR spectrum of **H**_**2**_ **L1**, two triplets (7.24 ppm and 7.39 ppm) and one doublet (7.54 ppm) were attributed to the protons of the aniline precursor ring (H16, H15, and H14; Figure 1). For ligand **H**_**2**_ **L2**, the signal of the H16 proton was not observed, and the positions of the signals for protons H15 and H14 are inverted due to the substantial inductive effect of the *p*-substituted chlorine atom of the aromatic ring. The signals of the pyrazine heterocyclic ring in the ^1^H-NMR spectra of ligands **H**_**2**_ **L1** and **H**_**2**_ **L2** were assigned as two doublets (8.62 ppm and 9.78 ppm) and a doublet of doublets (8.64 ppm) corresponding to protons H2, H5, and H3. M coupling with a small *J* value between H5 and H3 was observed. The protons of the methyl group (H8) bound to the carbon of the azomethine group gave a singlet at 2.45 ppm. The H10 and H12 protons and all of the protons of the carbon atoms of ligands **H**_**2**_ **L1** and **H**_**2**_ **L2** were assigned by the analysis of ^1^H-NMR, ^13^C-NMR, and DEPT135 and two-dimensional heteronuclear spectra (HSQC and HMBC) (see Supplementary Material S1).

#### 3.1.2. Single Crystal X-Ray Diffraction Studies

**Figure 2:**
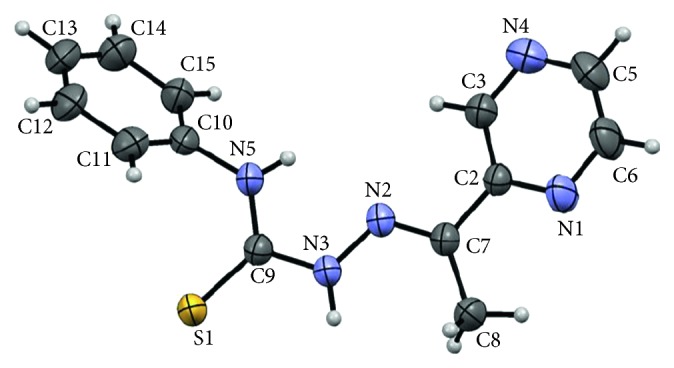
Molecular structure and atom labelling scheme for compound **H**_**2**_ **L1**.

The molecular structure of the ligand **H**_**2**_ **L1** (Figure 2) was determined by single crystal X-ray diffraction. **H**_**2**_ **L1** crystallizes in the triclinic space group *P*-1 with two molecules in the unit cell. As observed in the most significant bond lengths and angles of the compound (Table 2), in the solid state, **H**_**2**_ **L1** is present in the form of thione, showing structural parameters which reveal charge delocalisation between the atoms of the thiosemicarbazone group. In this context, it is important to note that the C-S bonds (C(9)-S(1)) are of 1.671(2) Å, which is slightly longer than a double C=S bond but shorter than a C-S bond. In addition, the N=N bonds (N(2)–N(3)) of the thiosemicarbazone group are of 1.373(3) Å, which is in agreement, if not somewhat longer than regular N=N bonds. Finally, the thiosermicarbazone group shows two types of C-N bonds: a shorter one between the carbon atom bound to the methyl group and one of the nitrogen atoms of the N=N moiety (N(2)-C(7)) with a value of 1.285(3) and a longer one between the other nitrogen atom of the N=N moiety and the carbon atom of the thione group (N(3)-C(9)) with a value of 1.364(3). This indicates the formation of a double bond between N(2) and C(7) as expected for the synthesized ligands.

The planarity of the thiosermicarbazone group is confirmed by the C-N-C, N-N-C, N-C-N, and N-C-S angles of the fragment which range between 113 and 127°, indicating a *sp* ^2^ character which support that the atoms C7, N2, N3, C9, and S1 are virtually located occupying the same meanplane with slight deviation of the planarity. In addition, the torsion angle N(2)-N(3)-C(9)-N(5) of ca. 7.3 supports the formation of the planar ligand.

**Figure 3:**
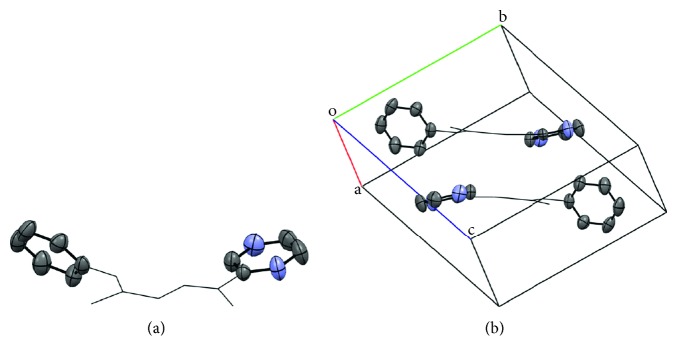
(a) Bent wings structure of **H**_**2**_ **L1**; (b) packing of **H**_**2**_ **L1** (only the atoms of the aromatic rings are depicted as ellipsoids for clarity).

Interestingly, the packing of the compound led to interesting observation in the arrangement of the aromatic ligands with reference to the meanplane formed by N(2)-N(3)-C(9)-N(5). Thus, the aromatic rings of the molecule deviate +15.9 (heterocycle) and +55.2° (phenyl) from the meanplane formed by N(2)-N(3)-C(9)-N(5) in **H**_**2**_ **L1** appearing as “bent wings” (Figure 3).

### 3.2. Synthesis and Characterisation of the Metal Complexes

**Scheme 2:**
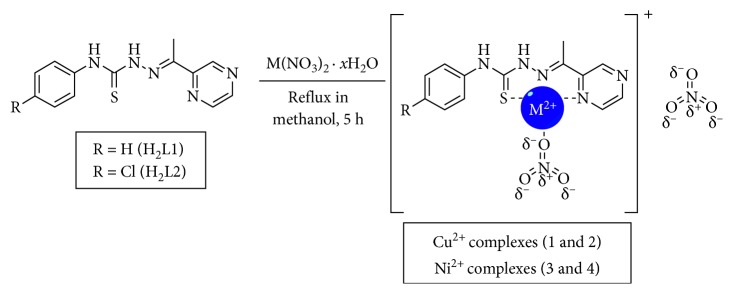
Obtaining copper and nickel complexes with thiosemicarbazone ligands.

The hydrated salts of copper(II) nitrates (for complexes **1** and **2**) and nickel(II) (for complexes **3** and **4**) were used to synthesise the metal complexes. The complexes were refluxed in a methanolic solution of ligands **H**_**2**_ **L1** and **H**_**2**_ **L2** with 1 : 1 metal-ligand stoichiometry with constant stirring for 5 hours (Scheme 2) [14, 29]. Each mixture was filtered, and the filtrates were concentrated and cooled to −4°C to afford brown solids corresponding to the coordination complexes (**1**–**4**). In all cases, the solids were soluble in DMSO and DMF and partially soluble in water, ethanol, and methanol.

**Figure 4:**
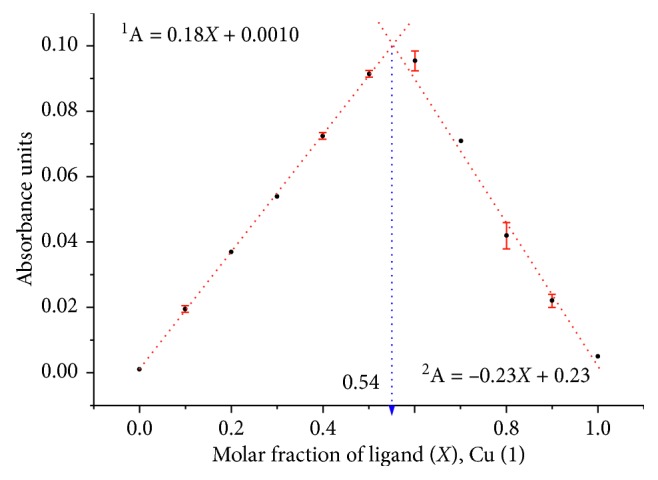
Method of continuous variation for complex **1** (as a representative example). In all cases, metals ions were allowed to react with TSC ligands for 15 h before absorbance was measured at the maximum of the absorbance of the complex (Cu = 410 nm; Ni = 403 nm). The assessment was performed with two independent stock solutions.

The obtained compounds were characterised using melting point determination, elemental analysis (C, H, and N), determination of the percentage of metal (Cu and Ni, by atomic absorption), IR spectroscopy, thermogravimetric analysis, and molar conductance measurements. To gain an insight into the stoichiometry of the metal complexes, the method of continuous variations (Job's method) was used (Figure 4). The plot shows the formation of 1 : 1 metal-ligand complexes. The results obtained from the different analytical techniques allowed to propose the structures of complexes **1**–**4**.

#### 3.2.1. Elemental Analysis, Melting Point, and Molar Conductance Measurements

The obtained **H**_**2**_ **L1** and **H**_**2**_ **L2** ligands and compounds **1**–**4** were isolated as air-stable crystalline solids with melting points above 200°C, and they did not undergo thermal decomposition at the temperatures tested. Microanalysis of the ligands and their respective metal complexes showed an excellent correlation with the proposed structures for **H**_**2**_ **L1** and **H**_**2**_ **L2** and complexes **1**–**4** with 1 : 1 [ML·NO_3_] stoichiometries (where L = tridentate TSC ligand). The error rate was less than 2% in almost all cases (Table 3).

Molar conductance measurements of the obtained complexes were performed in DMSO at a concentration of 1 × 10^−3^ mol·L^−1^ and 20°C. For complexes **1**–**4**, the molar conductance values ranged between 21.4 and 33.3 *μ*S·cm^−1^. These conductance values are consistent with the range reported for 1 : 1 electrolytes in this solvent [30], leading to the proposal of a structure with a single nitrate ion as the counterion, which was also confirmed after water solvation.

#### 3.2.2. IR Spectroscopy

In the infrared spectra of the ligands (**H**_**2**_ **L1** and **H**_**2**_ **L2**) and complexes **1**–**4**, absorption bands were observed at ∼3300 cm^−1^, 3150 cm^−1^, and 3050 cm^−1^, which were assigned to the vibrations of ν(O-H), ν(N-H10), and ν(N-H12) vibrations, respectively. Moreover, the absence of the band at 2600–2800 cm^−1^ and characteristic of the ν(C-SH) vibration indicate that, in the solid state, TSCs (**H**_**2**_ **L1** and **H**_**2**_ **L2**) act as neutral chelating ligands (thione tautomer) to form complexes **1**–**4**. The coordination of the Cu(II) and Ni(II) ions to the ligands through the azomethine group (C=N) was confirmed by the slight shift in the vibration to lower wavenumbers (1590 cm^−1^) compared to that of the free ligands (1615 cm^−1^). The band at ∼850 cm^−1^ assigned to the *ν* (C=S) vibration in the ligands was shifted to a lower wavenumber of ∼835 cm^−1^ in the complexes, indicating the sulphur was involved in coordination to the metal ion [14, 31, 32]. Thus, the TSCs act in a tridentate manner with an NNS system coordinating through the nitrogen atoms of the azomethine group and the pyrazine ring and the sulphur atom of the thioamide group. In order to clarify the metal ion effect on ligand, the IR spectra of the free ligand and its metal chelates were studied. The IR spectra of complexes **1**–**4** and the three bands (1434–1540 cm^−1^, 1306–1316 cm^−1^, and 1005–1023 cm^−1^) due to vibrational modes ν4, ν1, and ν2 of the nitrate group confirmed that this anion occupies the fourth coordination site in the monodentate mode [33]. The monodentate nature of the nitrate group in metal complexes with thiosemicarbazone ligands has been described through X-ray diffraction studies [34].

#### 3.2.3. Thermogravimetric Analysis

Thermal analysis provides important information regarding the stability and presence of water molecules in the crystalline network of compounds. The TG-DTG curves of the obtained complexes are shown in the supplementary information (see Supplementary Material S2). For complexes **1**–**3**, the decomposition process occurs in two steps, while for **4**, only one thermal decomposition event was observed. The results of the thermogravimetric analysis of compounds **1** and **2** showed a mass loss between 50 and 200°C, corresponding to the loss of the water molecules that may be present in the outer sphere of the complexes. With complex **3**, a mass loss was observed between 30 and 80°C due to the evaporation of methanol, which was the solvent used in the synthesis of this compound. In all cases, a mass loss was observed at temperatures above 250°C due to the formation of the respective Cu(II) and Ni(II) nitrates. The temperatures, mass loss, and products formed in each step in the decompositions of complexes **1**–**4** are reported in Table 4.

The results in Table 4 also show that the nickel compounds exhibit higher thermal stability, as they decompose at higher temperatures than the copper compounds, which agrees with the experimental melting temperatures (between 230 and 240°C for **1** and **2** and temperatures >300°C for complexes **3** and **4**).

### 3.3. DNA Interaction of Thiosemicarbazone Substitutes of 2-Acetylpyrazine and Their Complexes

Among other biological targets, such as proteins or RNA, DNA is usually the central objective of interaction of metallodrugs due to its importance in the control of cellular functions [35]. As a result, *cis*-platinum transition metal complexes have been used for developing novel therapeutic and diagnostic agents, which are used in chemotherapy treatments [36]. Studies on the interaction between DNA strands and metal complexes can explain the biological activity of coordination compounds since these interactions are one of the mechanisms that produce cytotoxic effects that cause cell death by apoptosis [37]. Therefore, several assays were performed to determine the mode of interaction between the synthesised compounds (ligands **H**_**2**_ **L1** and **H**_**2**_ **L2** and complexes **1**–**4**) and DNA using analytical methods such as UV-Vis spectroscopy, DNA viscosity measurements, and agarose gel electrophoresis.

#### 3.3.1. Electronic Absorption Monitoring Assays (UV-Vis Spectroscopy)

The analysis of the electronic absorption spectra is one of the most reliable methods for a preliminary evaluation of the interaction between metal complexes and DNA strands. These *in vitro* tests allow to obtain information about the strength and nature of these interactions [38]. In the present study, the electronic absorption spectra of the compounds synthesised (ligands **H**_**2**_ **L1** and **H**_**2**_ **L2** and complexes **1**–**4**) were recorded at a fixed concentration (20 *μ*mol·L^−1^) with increasing concentrations of CT-DNA (0 to 50 *μ*mol·L^−1^). The solutions of the complexes contained deionized water and DMSO approximately 0.1% to improve the solubilities of the compounds in the resuspension buffer.

**Figure 5:**
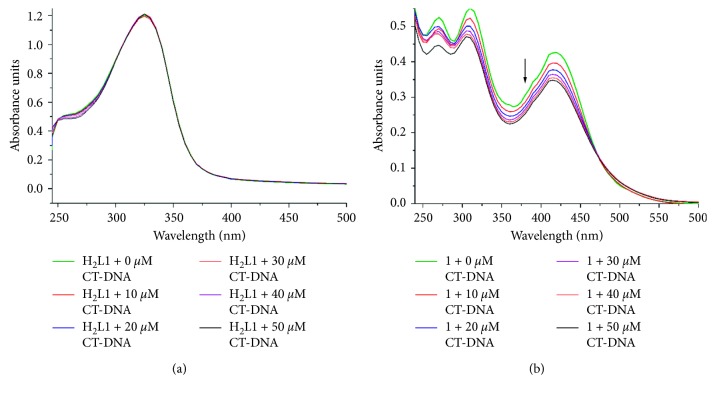
Electronic absorption spectra for (a) **H**_**2**_ **L1**; (b) **1** (as a representative example) at a constant concentration of 20 *μ*mol·L^−1^ titrated with CT-DNA to concentrations of 10 *μ*mol·L^−1^ (red), 20 *μ*mol·L^−1^ (blue), 30 *μ*mol·L^−1^ (violet), 40 *μ*mol·L^−1^ (orange), and 50 *μ*mol·L^−1^ (black) in nucleotides.

DNA is a biopolymer formed by nucleotides, and *π*-*π* interactions between nitrogenous bases prevail. Hypochromism and bathochromism in DNA are indicative of the intercalation mode, which is due to strong interactions between the compound and nitrogenous base pairs. These interactions produce a conformational change in the DNA structure, decreasing its exposure to radiation and, therefore, reducing its molar extinction coefficient [39, 40]. In the absorption spectra of the ligands (**H**_**2**_ **L1** and **H**_**2**_ **L2**), no significant changes were observed in the positions of the bands corresponding to the *n*⟶*π* ^*∗*^ and *π*⟶*π* ^*∗*^, electronic transitions, suggesting that these compounds exhibit groove binding interactions with CT-DNA (Figure 5). On the other hand, in the spectra of all the complexes, **1** to **4**, a gradual hypsochromic shift was observed in the *π*⟶*π* ^*∗*^ band with an increasing concentration of CT-DNA [41]. Metal ion coordination increases the rigidity of a compound, promoting its binding to the DNA strand through intercalation. Ligands can adopt spatial configurations in which the rotation of the nitrogen atom adjacent to the thiocarbonyl group disrupts molecular planarity, while the complex with a probable planar structure prevents free rotation, facilitating intercalation. In addition, the coupling between the *π* ^*∗*^ orbital of the aromatic component of the metal complexes with the *π* orbitals of the nitrogenous bases of the DNA is favoured, reducing the transition energy and decreasing the molar absorptivity. For complexes **1**–**4**, the intrinsic binding constant, *K*_b_, was determined from the spectral titration data using the following Wolfe–Shimmer equation [41]:

(1) $$\begin{array}{c} \frac{\left[\text{DNA}\right]}{\left(\varepsilon_{\text{a}}-\varepsilon_{\text{f}}\right)}=\frac{\left[\text{DNA}\right]}{\left(\varepsilon_{\text{b}}-\varepsilon_{\text{f}}\right)}+\frac{1}{K_{\text{b}}\left(\varepsilon_{\text{b}}-\varepsilon_{\text{f}}\right)}, \end{array}$$

where [DNA] is the concentration of CT-DNA in base pairs and the apparent absorption coefficients *ε*_a_, *ε*_f_, and *ε*_b_ are the apparent, free, and bound metal complex extinction coefficients, respectively. *K*_b_ is the binding constant in M^−1^, which was determined from the ratio of slope to intercept by plot of [DNA]/(*ε*_a_ − *ε*_f_) vs [DNA]. The binding constants for complexes **1**–**4** were 6.8 × 10^4^, 5.9 × 10^4^, 4.6 × 10^4^, and 3.9 × 10^4^ M^−1^, respectively (each experiment was performed in duplicate). The *K*_b_ of all the present complexes show a DNA binding affinity less than that for the proven classical intercalators like ethidium bromide (EthBr-DNA 7.7 × 10^7^ M^−1^) [41]. The hypsochromic behaviour exhibited by the compounds in the electronic absorption spectra is directly related to the type of interactions and binding constants. Therefore, a greater hypochromic effect indicates stronger interactions with the DNA strands. Copper complexes **1** and **2** showed the highest hypochromism (22% and 36%, respectively), and therefore, they presumably have a greater interaction with DNA, making them potential metallointercalators.

#### 3.3.2. Viscosity Measurements

**Figure 6:**
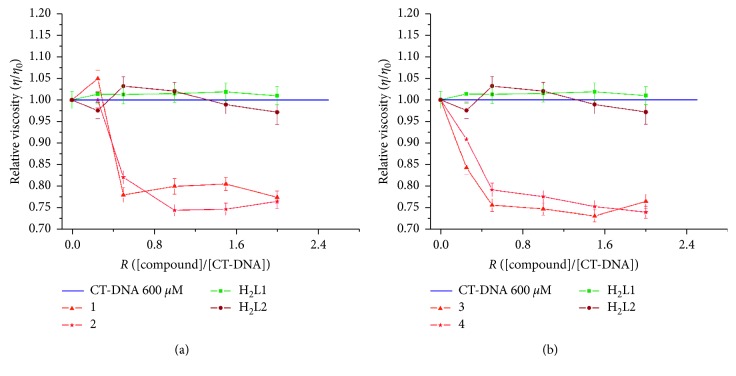
Effect of increasing amounts of compound (*R* = [compound]/[DNA]) between 0.2 and 2) on the relative viscosities of the CT-DNA solutions at 600 *s*M (blue line), **H**_**2**_ **L1** (green), and **H**_**2**_ **L2** (brown): (a) **1** and **2**; (b) **3** and **4**.

Structural alterations and variations in the DNA size due to its interactions with metallointercalators can be detected by viscosity measurements. In intercalation binding, the main effect is DNA elongation due to DNA unwinding, causing an increase in viscosity; in groove binding, chain elongation is less pronounced [42, 43]. To further clarify the mode of interaction between the complexes and DNA, viscosity measurements were carried out on samples of CT-DNA with a gradual increase in the concentration of the complexes (Figure 6).

The general trend for complexes **1**–**4** is that they reduce the relative viscosity of CT-DNA, indicating a predominantly covalent interaction between the metal complexes and the DNA strands. For the compounds tested, binding interactions may occur through a partial intercalative mode with CT DNA, where these partial intercalators could bend (or kink) the DNA helix, decreasing its effective length and in turn its viscosity. Thus, the results from viscosity measurements confirm the mode of DNA binding of the complexes, which are in agreement with the electronic absorption studies.

#### 3.3.3. Oxidative Cleavage Assays Monitored by Agarose Gel Electrophoresis

The biological activities of drugs can depend on their interactions with DNA, which can occasionally cause prolonged damage to DNA strands, resulting in cell death by apoptosis. Intermediate reagents involved in DNA cleavage are often reactive oxygen species (ROS) generated through redox reactions promoted by metal complexes. The interaction of these species with the nucleotides of the biopolymer can lead to direct cleavage of the double helix, conformational changes, or the formation of labile sites in DNA [44].

**Figure 7:**
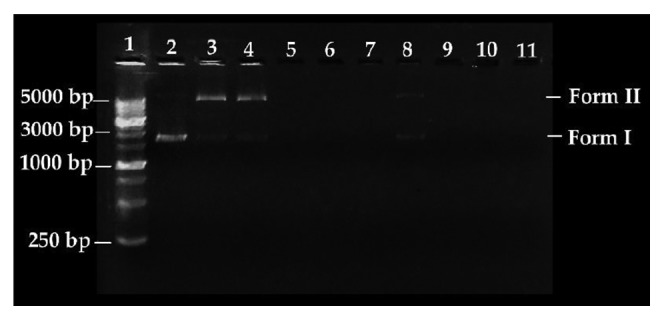
Agarose gel electrophoretic pattern of the pmCherry vector extracted from *E. coli* (5 ng·*μ*L) with H_2_O_2_ (100 *μ*mol·L^−1^) and varying concentrations of compounds **1** and **2**. Lane 1: HyperLadder 1 kb (15 *μ*L); Lane 2: pmCherry; Lane 3: pmCherry + H_2_O_2_; Lanes 4 and 8: pmCherry + compound (60 *μ*mol·L^−1^, **1** and **2**, respectively); Lanes 5 to 7 and 9 to 11: pmCherry + H_2_O_2_ + compound (25 *μ*mol·L^−1^, 50 *μ*mol·L^−1^, and 100 *μ*mol·L^−1^, **1** and **2**, respectively).

**Figure 8:**
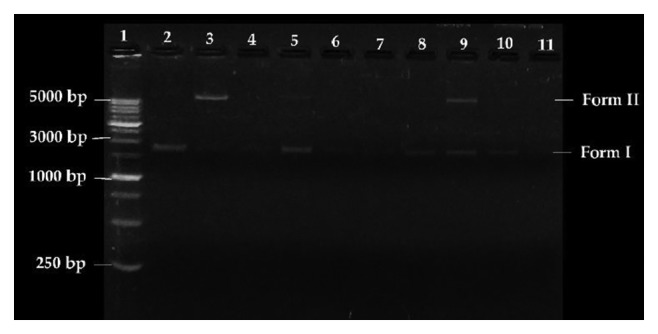
Agarose gel electrophoretic pattern of the pmCherry vector extracted from *E. coli* (5 ng·*μ*L) with H_2_O_2_ (100 *μ*mol·L^−1^) and varying concentrations of compounds **3** and **4**. Lane 1: HyperLadder 1 kb (15 *μ*L); Lane 2: pmCherry; Lane 3: pmCherry + H_2_O_2_; Lanes 4 and 8: pmCherry + compound (60 *μ*mol·L^−1^, **3** and **4**, respectively); Lanes 5 to 7 and 9 to 11: pmCherry + H_2_O_2_ + compound (25 *μ*mol·L^−1^, 50 *μ*mol·L^−1^, and 100 *μ*mol·L^−1^, **3** and **4**, respectively).

The effects of compounds **H**_**2**_ **L1** and **H**_**2**_ **L2** and complexes **1**–**4** on DNA conformation were evaluated by agarose gel electrophoresis, as shown in Figures 7 and 8. The DNA substrate was the pmCherry vector of *E. coli* BL21 (DH5*α*), which was obtained using standard protocols.

The electrophoretic mobilities of the different DNA conformations are indicative of the nuclease activity of the evaluated compounds. Form I corresponds to the supercoiled conformation, which has great mobility in the agarose gel. If a DNA strand is cleaved, it will change to a relaxed circular conformation (Form II), which has less mobility than Form I. Finally, Form III corresponds to the linear conformation caused by the cleavage of Form II, and this conformer migrates at an intermediate rate between Form I and Form II.

Compared to control DNA (Lane 2), complexes **1**–**4** at a concentration of 60 *μ*mol·L^−1^ can modify conformation of the supercoiled DNA (Form I) in the absence of external agents such as reducers or ROS produced by the molecular oxygen activator H_2_O_2_ (Lanes 4 and 8). In the presence of the ROS primer, complexes **1**–**4** (Lanes 5–7 and 9–11) exhibit increased nuclease activity and cause complete DNA cleavage. For the nickel complexes (**3** and **4**), this increase in activity is not as clear at the lowest concentration tested 25 *μ*mol·L^−1^ (Lanes 5 and 9, Figure 8), and a percentage of the relaxed circular conformation (Form II) is observed. In both cases, it was observed that oxidative DNA cleavage increases as the concentration of the complex increases.

To further elucidate the possible oxidative mechanism in DNA (vector pmCherry) induced by the complexes, a preliminary study was performed in the presence of hydroxyl radical scavengers (DMSO and KI). The results showed that the nuclease activities of complexes **1** to **4** are completely inhibited in the presence of DMSO or KI (no DNA conformational changes were observed), suggesting the possible involvement of hydroxyl radicals in oxidative DNA cleavage (see Supplementary Material S3).

### 3.4. Susceptibility of Bacteria to Thiosemicarbazone Substitutes of 2-Acetylpyrazine and Their Complexes

One of the main objectives in the design and synthesis of new antimicrobial agents is to find drug precursors that may act via different biological mechanisms to avoid the resistance developed by microorganisms and that minimise toxic effects in patients [15]. The antibacterial activities of the ligands (**H**_**2**_ **L1** and **H**_**2**_ **L2**) and complexes **1**–**4** were studied by microdilution against human pathogenic bacteria, including Gram-positive bacterial strains (*S. aureus* ATCC 25923, *L. monocytogenes* ATCC 19115, and *B*. *cereus* ATCC 10876) and Gram-negative bacterial strains (*E. coli* ATCC 25922, *S. typhimurium* ATCC 14028, and *K. pneumoniae* ATCC BAA-2146). The antibacterial drug ciprofloxacin was used as a standard control. The MICs are reported in Table 5.

In the experiments, all the studied compounds showed good activities against Gram-positive bacteria. Complexes **1**–**4** showed a significantly more potent antibacterial activities than were observed with the free ligands, indicating that the decrease in the polarity of the metal ions after coordination to the NNS donor system of TSCs increases the lipophilicity of the molecules, allowing them to more easily penetrate the lipid membranes and block essential enzymatic processes in the microorganisms [45]. The activities of the compounds are dependent on the concentration, with the best results being obtained for copper complexes **1** and **2** with MIC values of 3.9 *μ*g·mL^−1^ for *S. aureus* and *B. cereus* strains. The higher antibacterial activity in Cu(II) complexes, compared to the Ni(II) analogues, could be a consequence of redox processes for copper compounds, generating Cu(I) and Cu(0) species during the intracellular enzymatic reduction which increases the possibility of producing reactive oxygen species (ROS), which are highly associated with cellular death of pathogen microorganisms. As a result of the abovementioned results, these complexes are potential precursors of drugs that could be successfully used against common diseases caused by these bacteria, such as skin diseases, pneumonia, and nosocomial infections (*S. aureus*) as well as food-borne diseases (FBDs) (*B. cereus*). This analysis is consistent with what has been observed in DNA interaction assays in which nickel complexes **3** and **4** did not efficiently modify the conformation of the DNA double helix, which is one of the mechanisms of action of antimicrobial drugs. Moreover, the results showed that Gram-positive strains are more susceptible than Gram-negative strains to the synthesised compounds; therefore, it is possible to attribute the cytotoxic mechanism of action of the obtained compounds to the effects they have on the cell walls of microorganisms. When comparing the MIC values found for the Gram-negative and Gram-positive bacteria, no clear trend was observed in the antimicrobial activity based on the presence of an electrophilic substituent in the molecule. Copper and nickel nitrates did not show antibacterial activity in the range of concentrations tested.

## 4. Conclusions

In the present study, new copper(II) and nickel(II) complexes with tridentate thiosemicarbazone ligands **H**_**2**_ **L1** and **H**_**2**_ **L2** derived from 2-acetylpyrazine were synthesised. These compounds were characterised by different physicochemical techniques and spectroscopic methods. Single crystal X-ray diffraction studies revealed that, in the solid state, ligand **H**_**2**_ **L1** is present in the form of thione, showing bond distances which reveal charge delocalisation between the atoms of the thiosemicarbazone group. To evaluate the potential biological activities of the synthesised compounds, studies on their DNA interactions and antibacterial activities were performed. The results obtained from the electronic absorption spectra, viscosity measurements, and oxidative cleavage reactions showed that complexes **1**–**4** can efficiently interact with DNA strands. The antibacterial activity assays showed that the complexes have concentration-dependent bactericidal activities, and the best results were obtained for copper complexes **1** and **2** with MIC values of 3.9 *μ*g·mL^−1^ for *S. aureus* and *B. cereus* strains. These results are promising and contribute to our ongoing studies on the mechanisms of action of coordination complexes, and these results will inspire research on the design of new and better metallodrugs containing this kind of ligands.

## Figures and Tables

**Table 1:** Summarized crystallographic data.

Compound	Melting point (°C)
Empirical formula	C_13_H_13_N_5_S
*M*_r_ (g·mol^−1^)	271.34
Temperature (K)	298
Crystal system	Triclinic
Space group	*P* − 1
Crystal size (mm)	0.100 × 0.250 × 0.348
*a* (Å)	5.8565 (2)
*b* (Å)	10.0980 (4)
*c* (Å)	11.4853 (4)
*α* (°)	77.2761 (13)
*β* (°)	86.6013 (12)
*γ* (°)	89.7176 (12)
*V* (Å^3^)	661.36 (4)
*Z*	2
*θ* range	2.44 to 25.35
*D*_calc_ (kg·m^−3^)	1.363
Refined parameters	179
Total reflections	2391
Unique reflections	1875
*R*1 [*I* > 2*σ*(*I*)]	0.0548
*wR*_2_	0.1436
Goodness of fit	1.071

**Table 2:** Selected bond lengths and angles of H_2_L1.

Bond or angles	H_2_L1
C(9)-S(1)	1.671(2)
N(2)-N(3)	1.373(3)
N(2)-C(7)	1.285(3)
N(3)-C(9)	1.364(3)
N(5)-C(9)	1.337(3)
C(7)-N(2)-N(3)	118.9(2)
N(2)-N(3)-C(9)	118.5(2)
N(3)-C(9)-N(5)	114.8(2)
N(3)-C(9)-S(1)	119.6(2)
N(5)-C(9)-S(1)	125.5(2)
Torsion N(2)-N(3)-C(9)-N(5)	7.3(2)

**Table 3:** Melting points and elemental analysis of the obtained compounds.

Compound	m.p.	%H	%C	%N	%S	%M
**H**_**2**_ **L1**	206	4.75/4.83	57.12/57.54	25.16/25.81	10.75/11.82	—
**H**_**2**_ **L2**	208	3.87/3.96	50.62/51.06	22.77/22.90	10.12/10.48	—
**1**	232	3.35/3.73	30.28/30.44	18.88/19.12	6.14/6.25	12.19/12.39
**2**	240	3.10/2.76	32.03/30.54	20.31/19.17	5.32/6.27	12.56/12.43
**3**	312	3.28/3.53	35.13/34.59	19.31/20.17	7.90/6.60	12.26/12.07
**4**	327	2.71/2.48	32.88/31.96	19.50/20.07	6.98/6.56	11.86/12.02

m.p.: melting point (°C); values are %(found/calculated); M: **1**–**2** (Cu) and **3**–**4** (Ni).

**Table 4:** Experimental values of the temperature and percentage of mass loss observed in each step of the TG-DTG curves in an inert atmosphere.

Compound	Temperature °C (%) mass loss		Products
	First step	Second step	First step Second step
**1**	50–200/10.8 (calcd. 10.5%)	220–250/62.1 (calcd. 63.4%)	[Cu(1)(NO_3_)]NO_3_ Cu(NO_3_)_2_
**2**	50–200/3.89 (calcd. 3.52%)	230–270/62.7 (calcd. 63.3%)	[Cu(2)(NO_3_)]NO_3_ Cu(NO_3_)_2_
**3**	30–120/6.11 (calcd. 6.59%)	250–500/50.8 (calcd. 49.7%)	[Ni(1)(NO_3_)]NO_3_ Ni(NO_3_)_3_
**4**	280–320/47.7 (calcd. 49.9%)	—	Ni(NO_3_)_3_

Note: calcd., % mass loss calculated.

**Table 5:** Antibacterial activities of the studied compound.

Compound	MIC (*μ*g·mL^−1^)					
	*S. aureus*ATCC 25923	*L. monocytogenes*ATCC 19115	*B. cereus*ATCC 10876	*E. coli*ATCC 25922	*S. typhimurium*ATCC 14028	*K. pneumoniae*ATCC BAA-2146
**Cu(NO**_**3**_ **)**_**2**_	>2000	>2000	>2000	>2000	>2000	>2000
**Ni(NO**_**3**_ **)**_**2**_	>2000	>2000	>2000	>2000	>2000	>2000
**H**_**2**_ **L1**	500	1000	500	500	1000	2000
**H**_**2**_ **L2**	500	2000	500	>2000	2000	>2000
**1**	3.9	15.5	3.9	63	125	500
**2**	3.9	31.3	3.9	125	2000	>2000
**3**	63	125	125	1000	>2000	>2000
**4**	125	125	125	1000	>2000	2000
**Cip**	0.5	0.5	0.5	<0.008	0.125	>4
**AgNO**_**3**_	<100	<100	<100	<100	<100	<100

MIC: minimum inhibitory concentration; Cip: ciprofloxacine; AgNO_3_: reference/medication control.

## Data Availability

The data used to support the findings of this study are available from the corresponding author upon request.

## Supplementary Materials

Supplementary Materials S1: copies of ^1^H-NMR, ^13^C-NMR, DEPT135, and two-dimensional heteronuclear spectra (HSQC and HMBC) for **H**_**2**_ **L1** and **H**_**2**_ **L2**; S2: TG-DTG curves of the complexes obtained (**1**–**4)**; S3: electrophoretic pattern of the pmCherry vector in the presence of scavengers.
