# Supplementary figures and images for: Cu(II) and Ni(II) Complexes with New Tridentate NNS Thiosemicarbazones: Synthesis, Characterisation, DNA Interaction, and Antibacterial Activity
Source: Bioinorg Chem Appl. 2019 Jul 1;2019:3520837. doi: 10.1155/2019/3520837 (PMC6636485; doi:10.1155/2019/3520837)

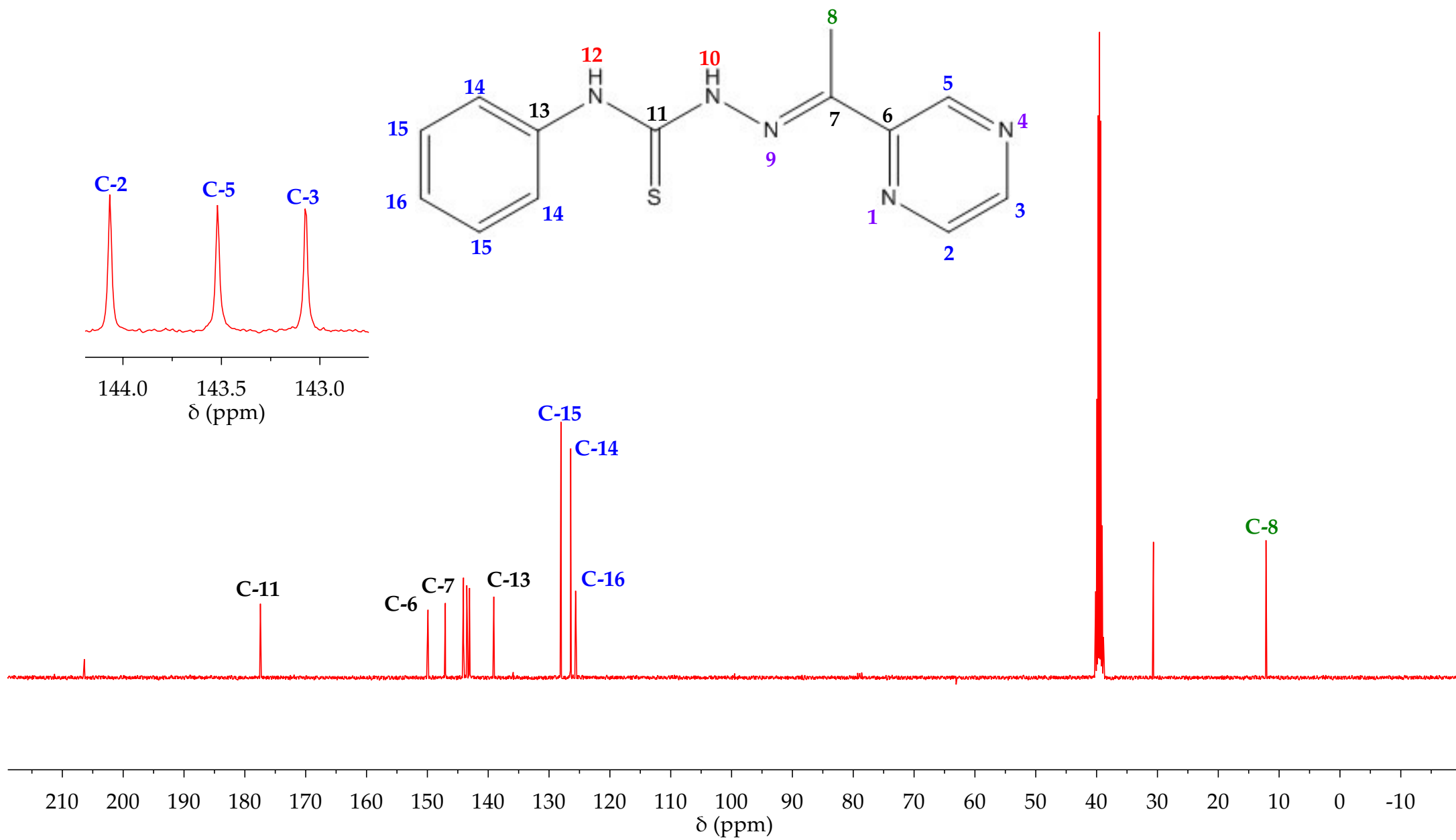

Supplement: Supplementary Materials — S1: copies of 1H-NMR, 13C-NMR, DEPT135, and two-dimensional heteronuclear spectra (HSQC and HMBC) for H 2 L1 and H 2 L2; S2: TG-DTG curves of the complexes obtained (1–4); S3: electrophoretic pattern of the pmCherry vector in the presence of scavengers. [file 3520837.f1.zip › 3520837.f1/S1. NMR/1. H2L1 13C NMR.pdf]

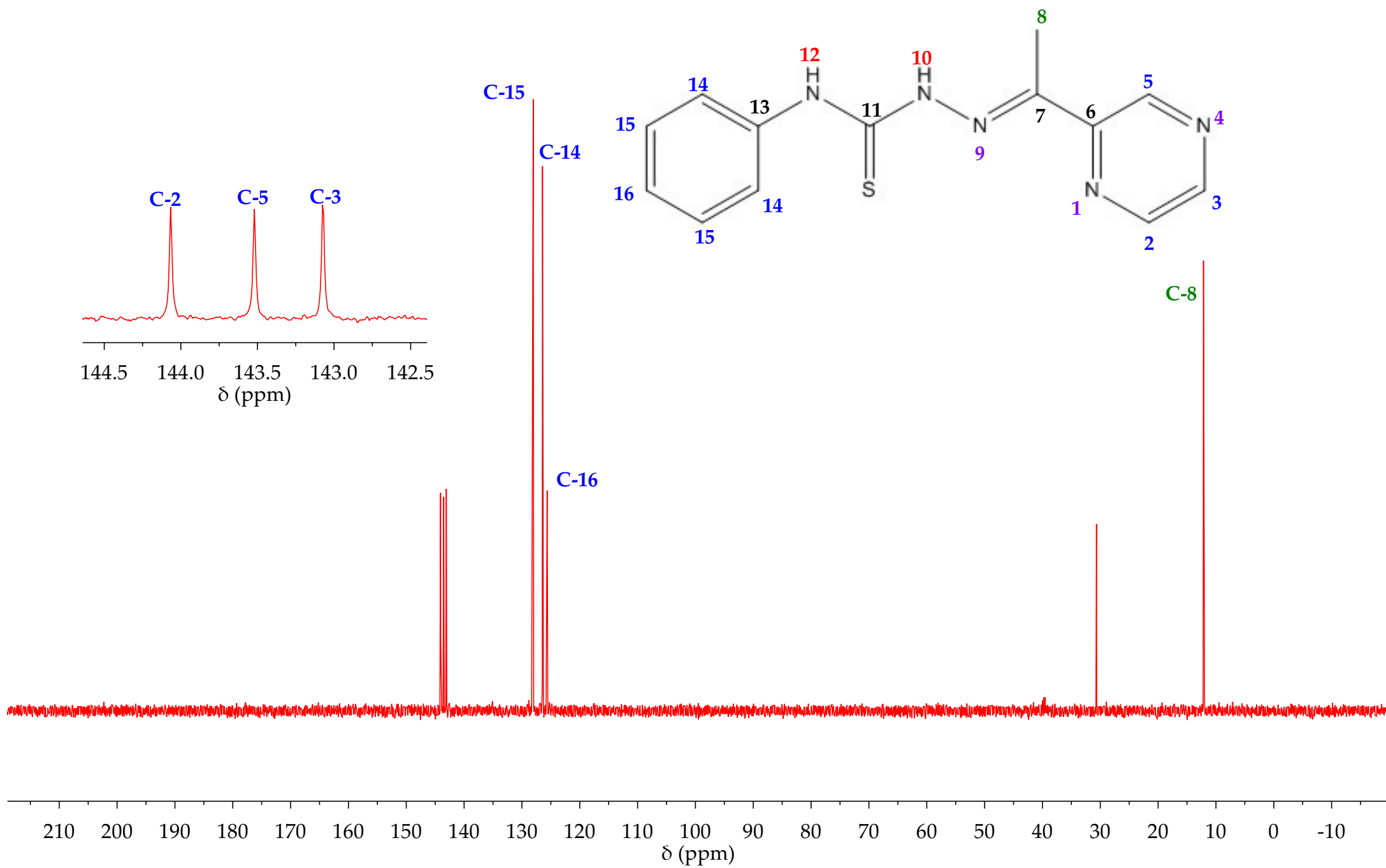

Supplement: Supplementary Materials — S1: copies of 1H-NMR, 13C-NMR, DEPT135, and two-dimensional heteronuclear spectra (HSQC and HMBC) for H 2 L1 and H 2 L2; S2: TG-DTG curves of the complexes obtained (1–4); S3: electrophoretic pattern of the pmCherry vector in the presence of scavengers. [file 3520837.f1.zip › 3520837.f1/S1. NMR/2. H2L1 DEPT135 NMR.pdf]

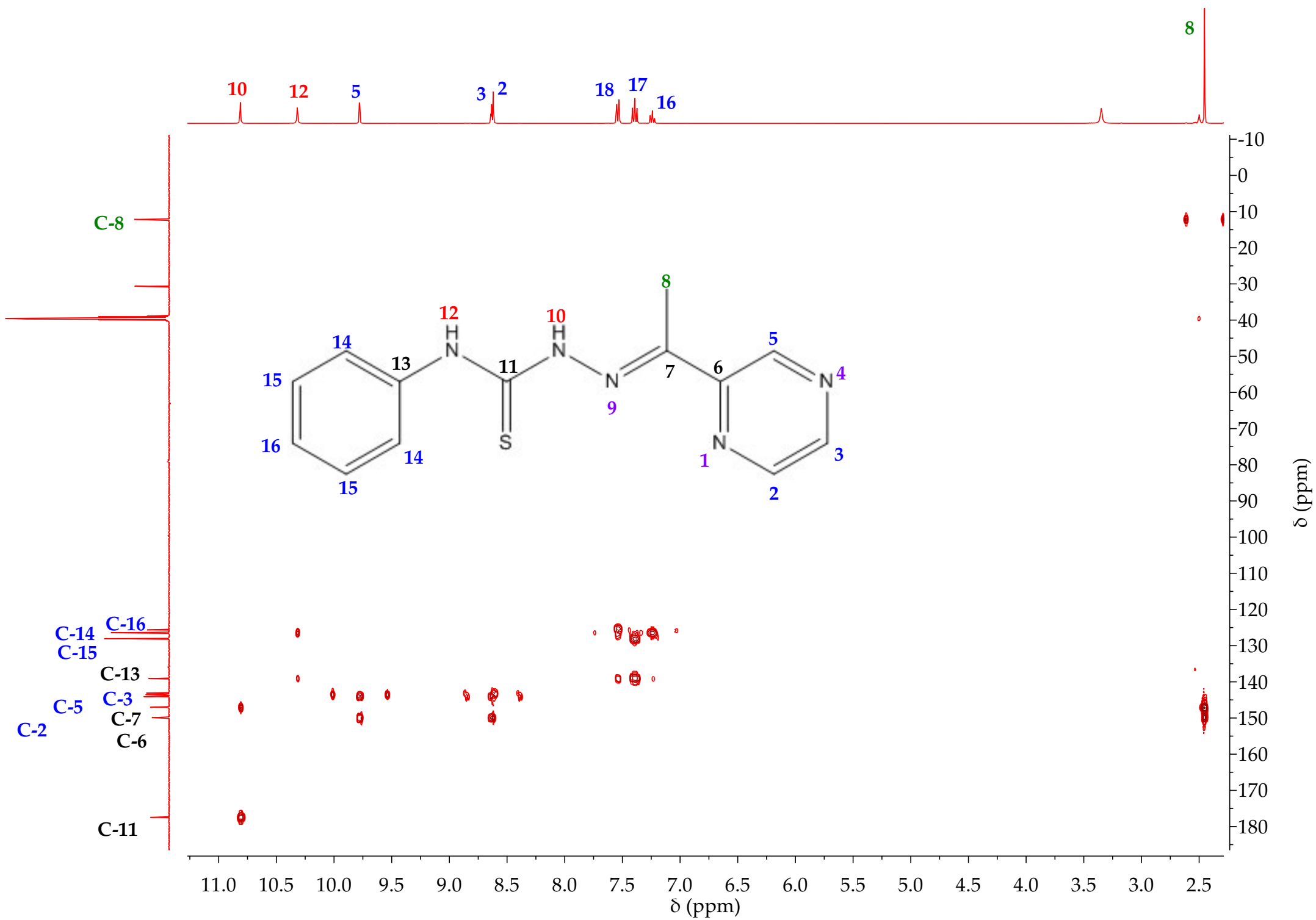

Supplement: Supplementary Materials — S1: copies of 1H-NMR, 13C-NMR, DEPT135, and two-dimensional heteronuclear spectra (HSQC and HMBC) for H 2 L1 and H 2 L2; S2: TG-DTG curves of the complexes obtained (1–4); S3: electrophoretic pattern of the pmCherry vector in the presence of scavengers. [file 3520837.f1.zip › 3520837.f1/S1. NMR/3. H2L1 HMBC NMR.pdf]

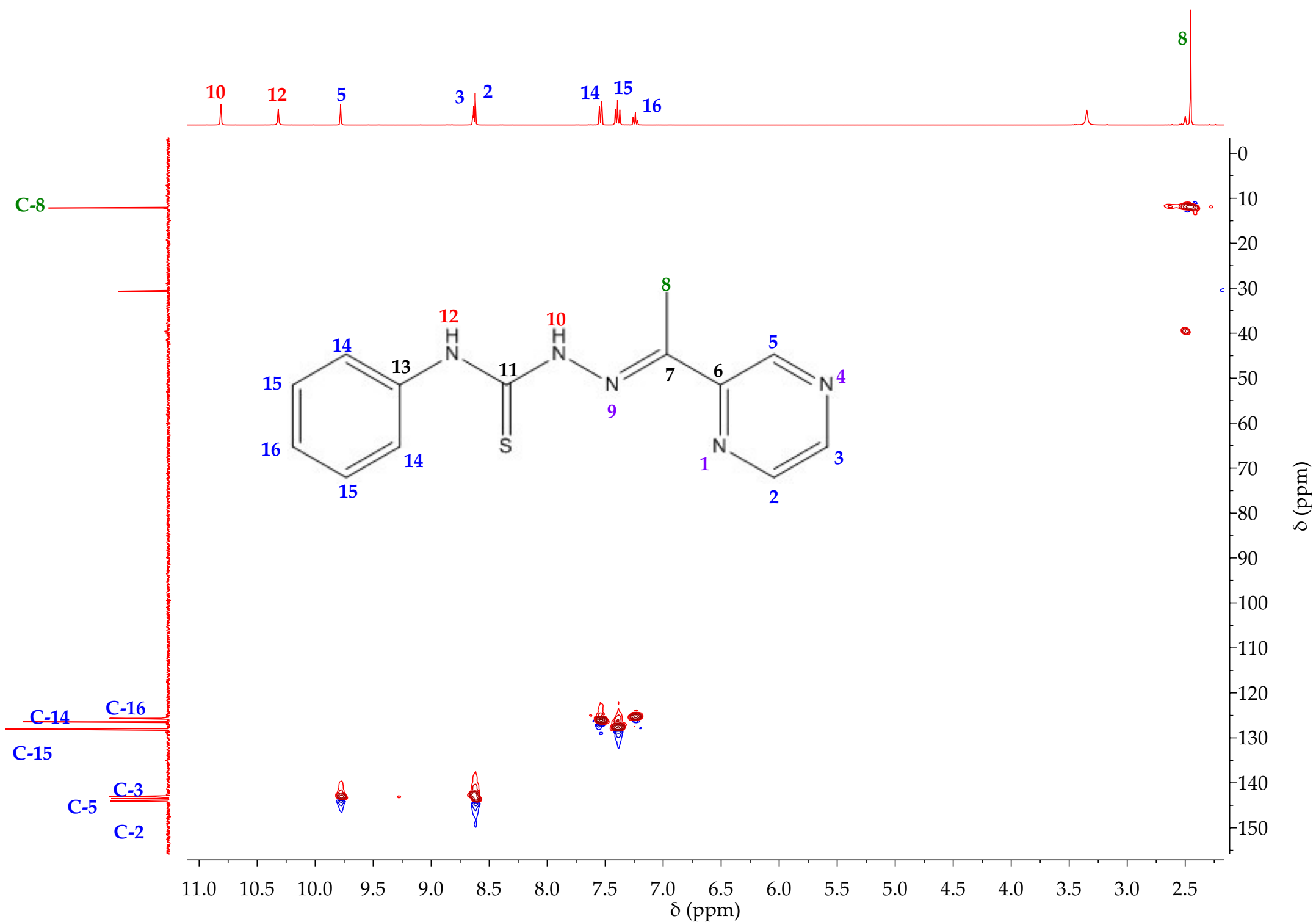

Supplement: Supplementary Materials — S1: copies of 1H-NMR, 13C-NMR, DEPT135, and two-dimensional heteronuclear spectra (HSQC and HMBC) for H 2 L1 and H 2 L2; S2: TG-DTG curves of the complexes obtained (1–4); S3: electrophoretic pattern of the pmCherry vector in the presence of scavengers. [file 3520837.f1.zip › 3520837.f1/S1. NMR/4. H2L1 HSQC NMR.pdf]

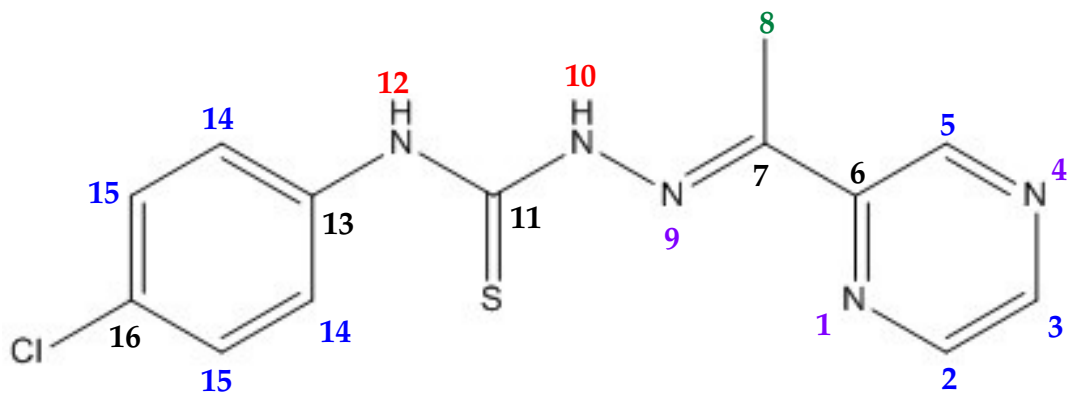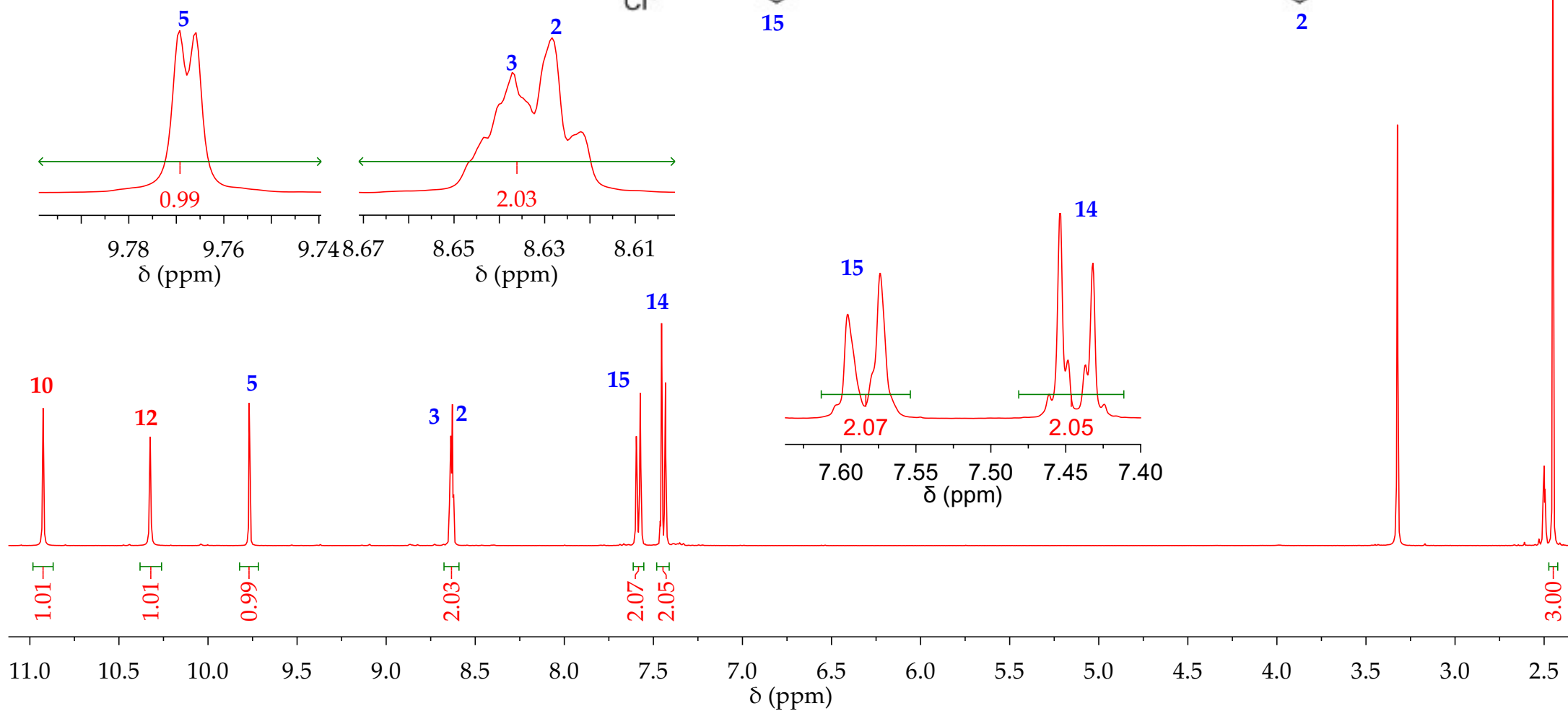

Supplement: Supplementary Materials — S1: copies of 1H-NMR, 13C-NMR, DEPT135, and two-dimensional heteronuclear spectra (HSQC and HMBC) for H 2 L1 and H 2 L2; S2: TG-DTG curves of the complexes obtained (1–4); S3: electrophoretic pattern of the pmCherry vector in the presence of scavengers. [file 3520837.f1.zip › 3520837.f1/S1. NMR/5. H2L2 1H NMR.pdf]

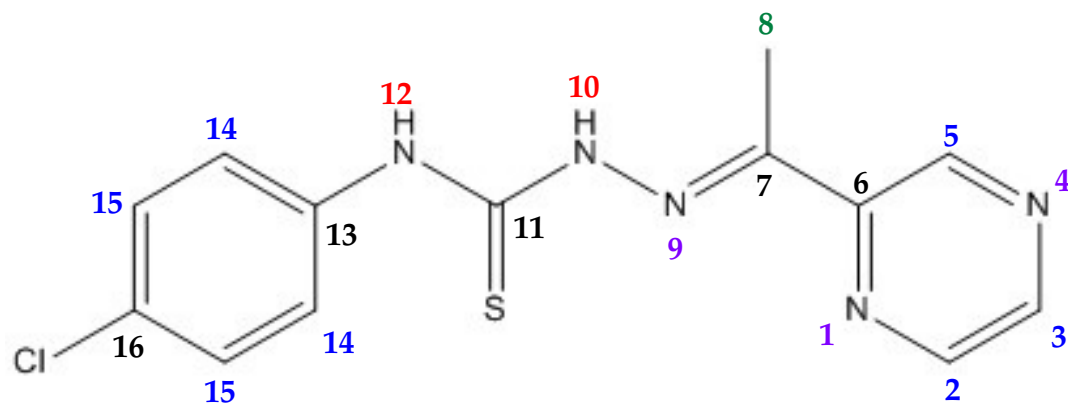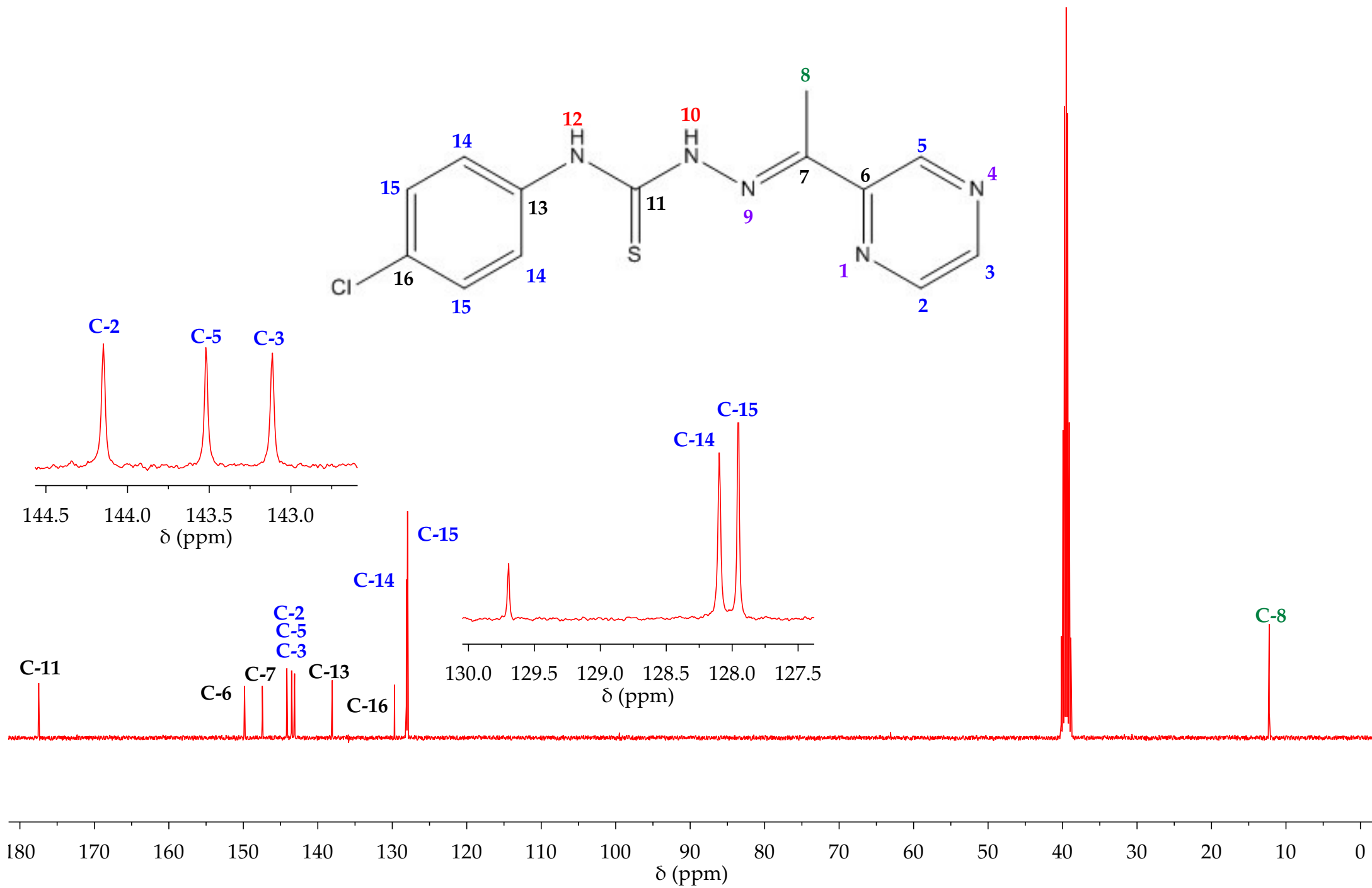

Supplement: Supplementary Materials — S1: copies of 1H-NMR, 13C-NMR, DEPT135, and two-dimensional heteronuclear spectra (HSQC and HMBC) for H 2 L1 and H 2 L2; S2: TG-DTG curves of the complexes obtained (1–4); S3: electrophoretic pattern of the pmCherry vector in the presence of scavengers. [file 3520837.f1.zip › 3520837.f1/S1. NMR/6. H2L2 13C NMR.pdf]

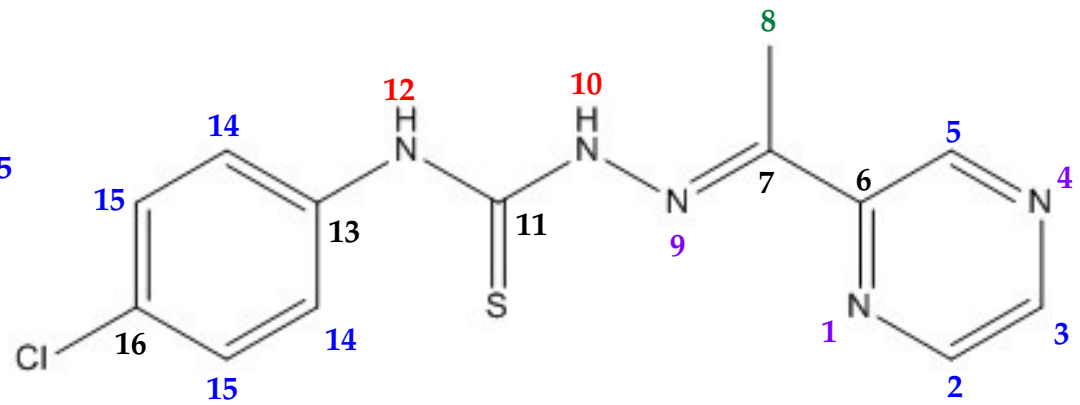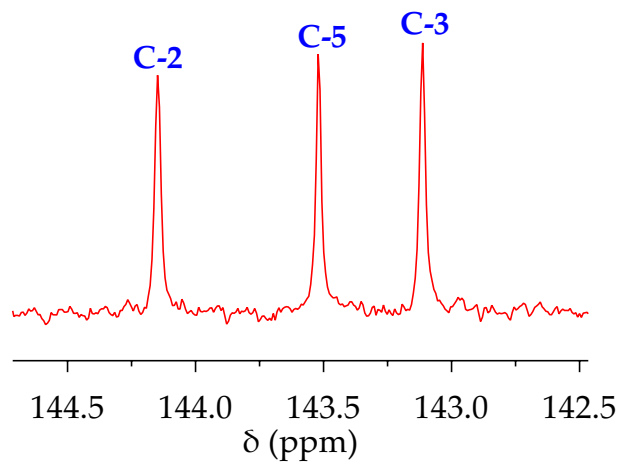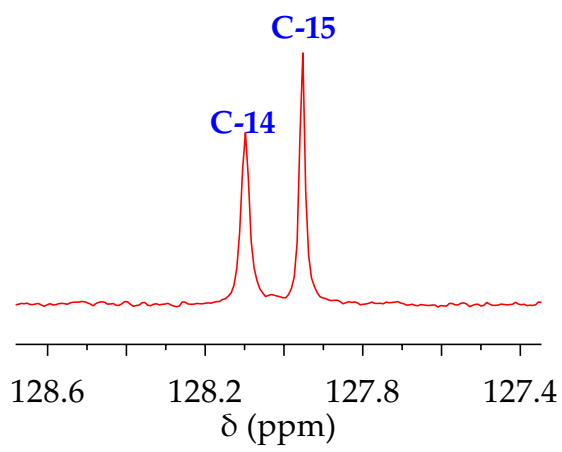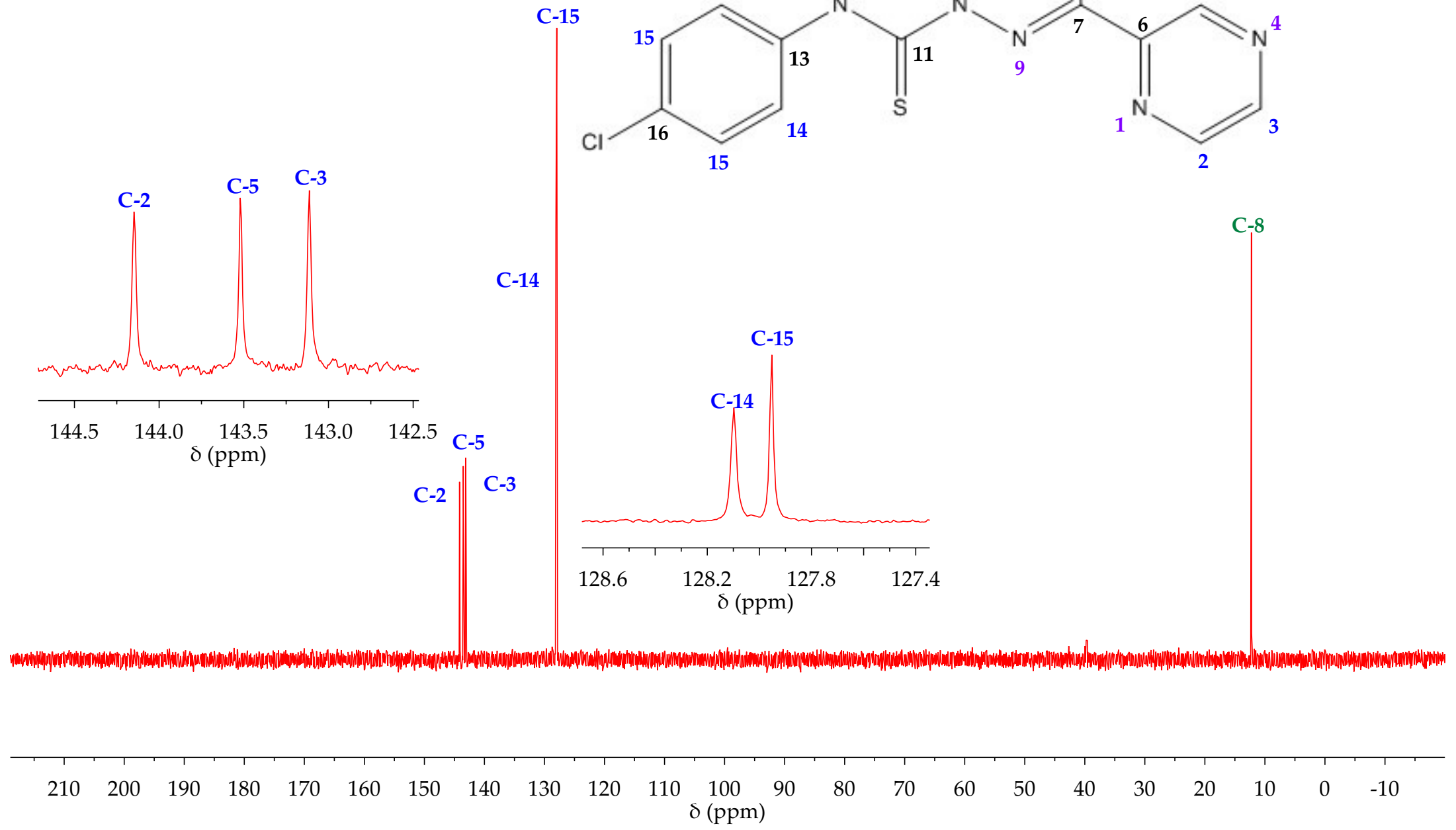

Supplement: Supplementary Materials — S1: copies of 1H-NMR, 13C-NMR, DEPT135, and two-dimensional heteronuclear spectra (HSQC and HMBC) for H 2 L1 and H 2 L2; S2: TG-DTG curves of the complexes obtained (1–4); S3: electrophoretic pattern of the pmCherry vector in the presence of scavengers. [file 3520837.f1.zip › 3520837.f1/S1. NMR/7. H2L2 DEPT135 NMR.pdf]

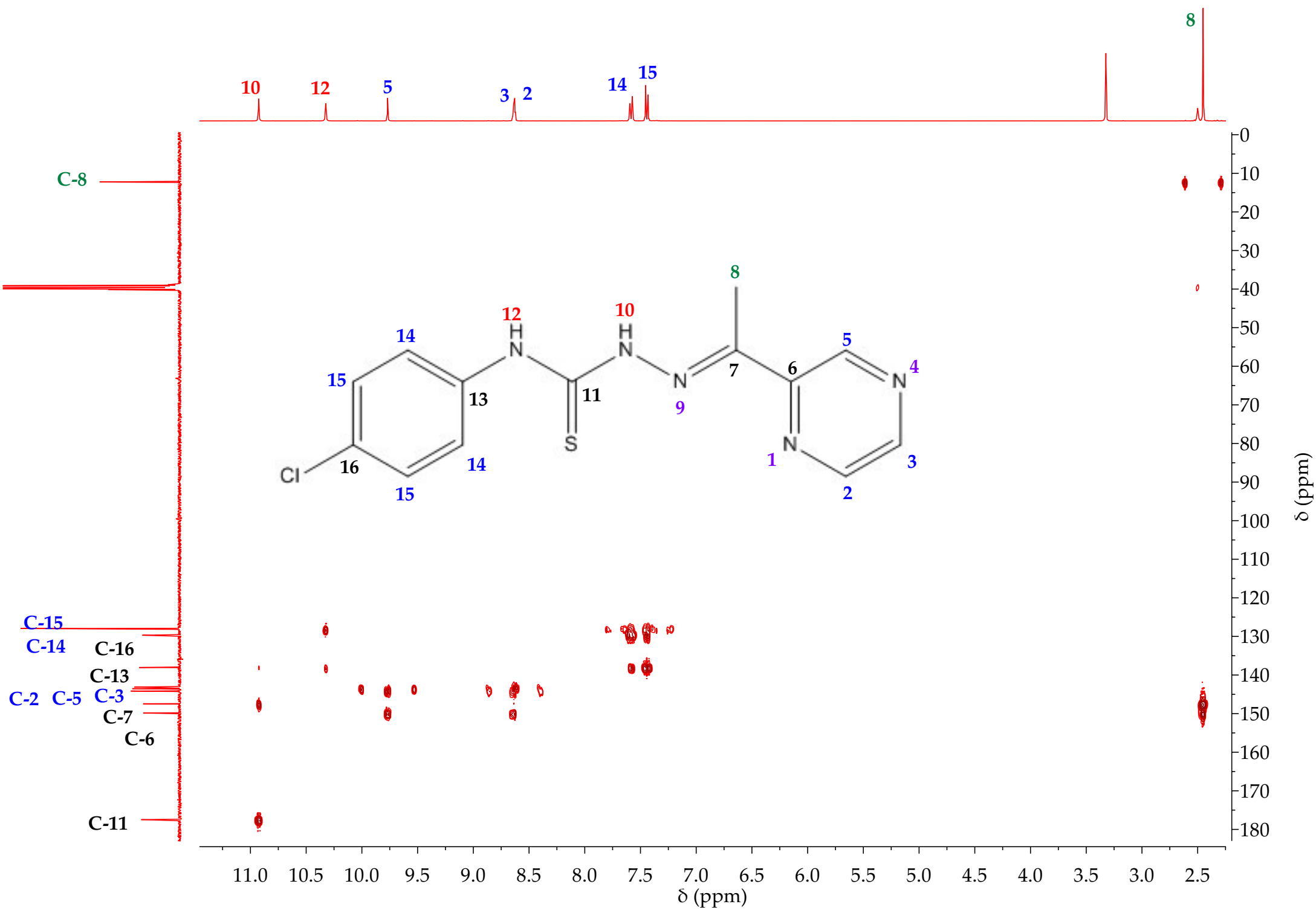

Supplement: Supplementary Materials — S1: copies of 1H-NMR, 13C-NMR, DEPT135, and two-dimensional heteronuclear spectra (HSQC and HMBC) for H 2 L1 and H 2 L2; S2: TG-DTG curves of the complexes obtained (1–4); S3: electrophoretic pattern of the pmCherry vector in the presence of scavengers. [file 3520837.f1.zip › 3520837.f1/S1. NMR/8. H2L2 HMBC NMR.pdf]

C-8

C-15

C-14

C-3

C-5

C-2

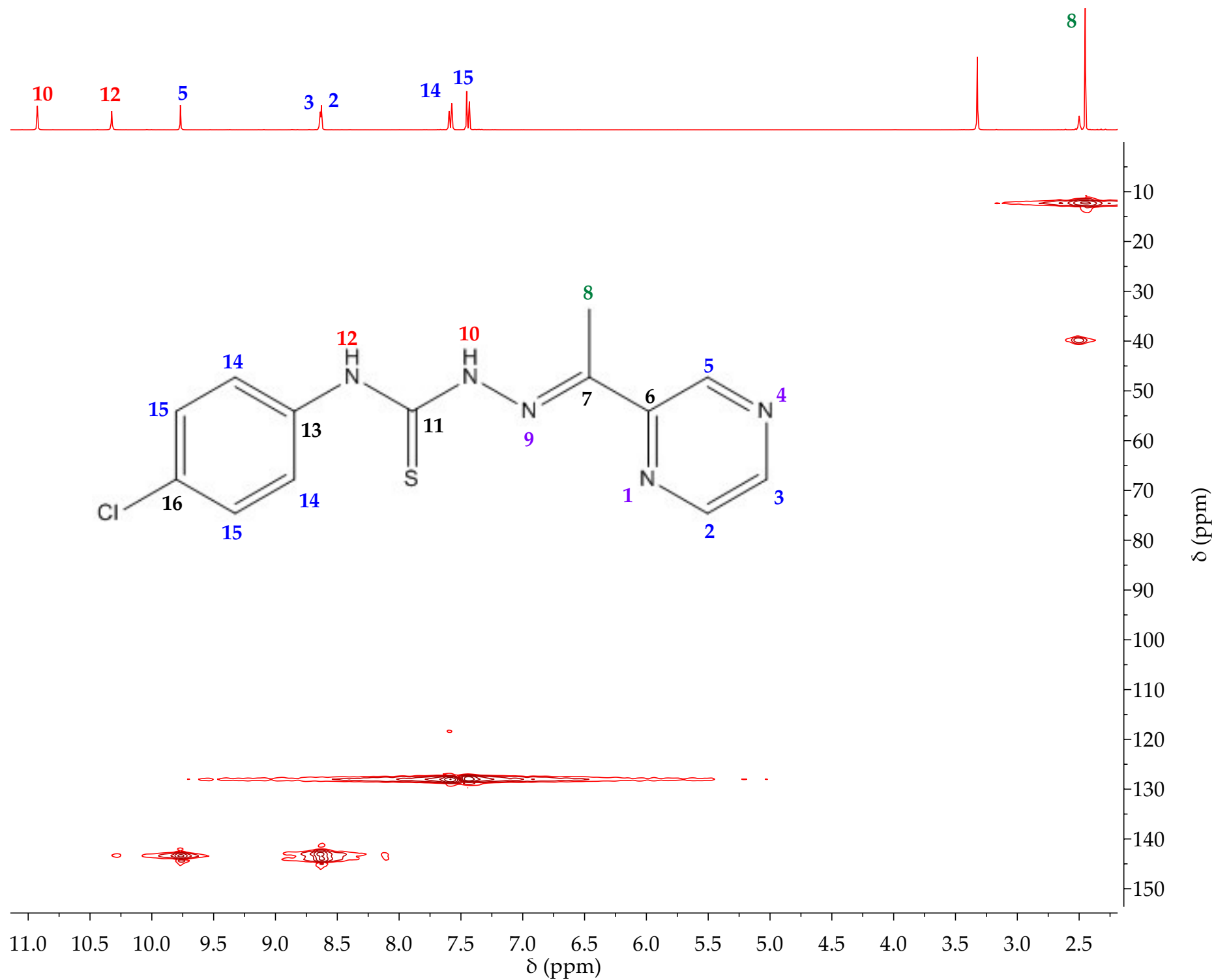

Supplement: Supplementary Materials — S1: copies of 1H-NMR, 13C-NMR, DEPT135, and two-dimensional heteronuclear spectra (HSQC and HMBC) for H 2 L1 and H 2 L2; S2: TG-DTG curves of the complexes obtained (1–4); S3: electrophoretic pattern of the pmCherry vector in the presence of scavengers. [file 3520837.f1.zip › 3520837.f1/S1. NMR/9. H2L2 HSQC NMR.pdf]

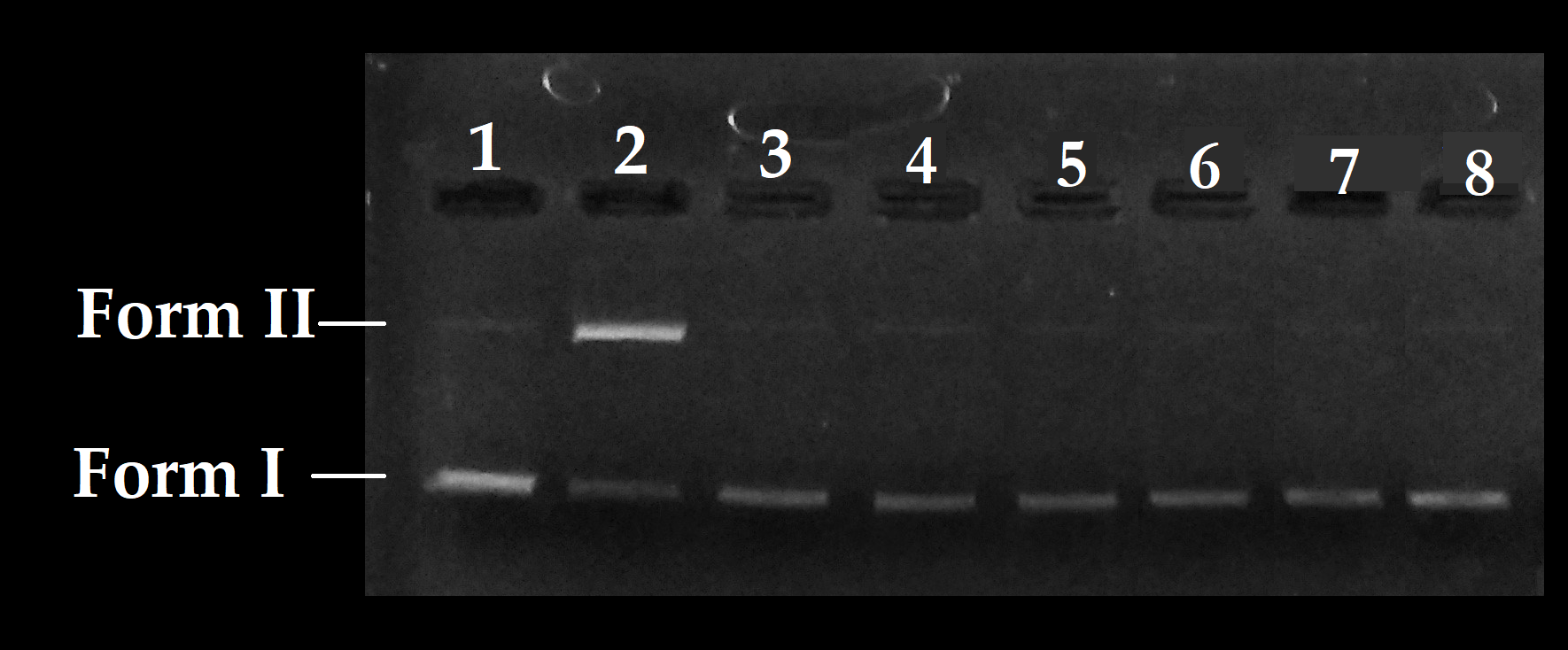

Supplement: Supplementary Materials — S1: copies of 1H-NMR, 13C-NMR, DEPT135, and two-dimensional heteronuclear spectra (HSQC and HMBC) for H 2 L1 and H 2 L2; S2: TG-DTG curves of the complexes obtained (1–4); S3: electrophoretic pattern of the pmCherry vector in the presence of scavengers. [file 3520837.f1.zip › 3520837.f1/S3.Electrophoretic pattern of pmCherry vector in the presence of scavengers.png]
